# The wild taxa utilized as vegetables in Sicily (Italy): a traditional component of the Mediterranean diet

**DOI:** 10.1186/s13002-018-0215-x

**Published:** 2018-02-14

**Authors:** Anna Geraci, Filippo Amato, Giuseppe Di Noto, Giuseppe Bazan, Rosario Schicchi

**Affiliations:** 1 Dipartimento di Scienze e Tecnologie Biologiche Chimiche e Farmaceutiche (STEBICEF), Sezione di Botanica ed Ecologia Vegetale, Università degli Studi di Palermo, Via Archirafi 38, 90123 Palermo, Italy; 2 ARPA Sicilia ST Palermo UO Monitoraggi Ambientali, Via Nairobi, 4, 90129 Palermo, Italy; 3 Dipartimento Regionale dello Sviluppo Rurale e Territoriale, Via regione siciliana, 4600 Palermo, Italy; 4 Dipartimento di Scienze Agrarie, Alimentari e Forestali (SAAF), Università degli Studi di Palermo, Viale delle Scienze Ed. 4, I-90128 Palermo, Italy

**Keywords:** Ethnobotany, Biocultural diversity, Traditional knowledge, Rural cultural heritage, Traditional agroecosystems

## Abstract

**Background:**

Wild vegetables in the Mediterranean Basin are still often consumed as a part of the diet and, in particular, there is a great tradition regarding their use in Sicily.

In this study, an ethnobotanical field investigation was carried out to (a) identify the wild native taxa traditionally gathered and consumed as vegetables in Sicily, comparing the collected ethnobotanical data with those of other countries that have nominated the Mediterranean diet for inclusion in the UNESCO Representative List of the Intangible Cultural Heritage of Humanity and (b) highlight new culinary uses of these plants.

**Methods:**

Interviews were carried out in 187 towns and villages in Sicily between 2005 and 2015. A total of 980 people over the age of 50 were interviewed (mainly farmers, shepherds, and experts on local traditions).

Plants recorded were usually collected in collaboration with the informants to confirm the correct identification of the plants. The frequencies of citation were calculated.

**Results:**

Two hundred fifty-three taxa (specific and intraspecific) belonging to 39 families, and 128 genera were recorded (26 were cited for the first time). The most represented families were Asteraceae, Brassicaceae, Apiaceae, Amaryllidaceae, Malvaceae, and Polygonaceae. Only 14 taxa were cited by 75% of the people interviewed.

The aerial parts of wild plants, including leaves, tender shoots, and basal rosettes, are the main portions collected, while the subterranean parts are used to a lesser extent. For some vegetables, more parts are utilized. Most of the reported vegetables are consumed cooked.

In addition to the widely known vegetables (*Borago officinalis*, *Beta* spp., *Cichorium* spp., *Brassica* spp., *Carduus* spp., etc.), the so-called ancient vegetables are included (*Onopordum illyricum*, *Centaurea calcitrapa*, *Nasturtium officinale*, *Scolymus* spp., *Smyrnium rotundifolium*), and some unique uses were described.

Comparing the Sicilian findings to those from other countries, a very high number of vegetable taxa were detected, 72 of which are eaten only in Sicily, while 12 are consumed in all the Mediterranean countries examined.

**Conclusions:**

The research shows a high level of Sicilian knowledge about using wild plants as a traditional food source. Wild vegetables are healthy and authentic ingredients for local and ancient recipes, which are fundamental to the revitalization of quality food strictly connected to traditional agroecosystems.

## Background

The Mediterranean diet represents the dietary pattern usually applied among the populations living closest to the Mediterranean Sea; it has been extensively reported to be a model of healthy eating for its contribution to a favorable health status and better quality of life and has been recognized on the UNESCO Representative List of the Intangible Cultural Heritage of Humanity for Italy, Spain, Portugal, Morocco, Greece, Cyprus, and Croatia [1–4]. Several studies in different populations have established the beneficial roles of the main components of the Mediterranean diet in preventing cardiovascular and chronic degenerative diseases [5–12]. The characteristics of this diet are “abundant plant foods, fresh fruit as the typical daily dessert, olive oil as the principal source of fat, dairy products (principally cheese and yogurt), and fish and poultry consumed in low to moderate amounts, zero to four eggs consumed weekly, red meat consumed in low amounts, and wine consumed in low to moderate amounts, normally with meals” [13, 14]. The daily and abundant consumption of vegetables (including wild ones), fresh fruits, and cereals together with the habitual use of olive oil guarantees a high intake of monounsaturated fatty acids, carotenoids, ascorbic acid and other vitamins, tocopherols, minerals, and several healthy substances, such as polyphenols and anthocyanins [15–17]. Moreover, vegetables are also very important for the intake of dietary fiber, which improves intestinal peristalsis and reduces the glycaemic index of a meal [18]. A high level of vegetable consumption produces an overall positive effect on human health [19–22].

Wild vegetables, those that grow spontaneously without being cultivated (including native species and some introduced taxa that have become naturalized), in the Mediterranean Basin are still widely consumed as part of the diet; they represent a new trend in nutrition in contemporary European cuisine because of their health benefits [23–27]. These plants have been an important part of the common daily diet in the Mediterranean and the Near East for millennia, but only recently has there been an increase in international literature focusing on the identification and the traditional uses of gathered wild vegetables for Mediterranean countries such as Croatia [28–30], Herzegovina [31], Turkey [32–37], Cyprus [38], Greece (including Crete) [39, 40], Italy [41–62], Spain [63–72], and Morocco [73, 74]. In the Mediterranean region, the use of wild vegetables is strictly linked to the traditional cuisine of each country, and it includes the traditional knowledge about cooking methods and the particular events at which they are consumed.

Wild vegetables play a very important role in the diet of the people living in Sicily, an island located in the middle of the Mediterranean region. In the past, people used to go almost daily, especially during the winter and spring, to the countryside and the margins of cultivated fields and woods, looking for wild vegetables to eat. This alimentary habit derived substantially from the situation of poverty in which most of the rural and urban population lived [75]. In the last 40 years, the eating habits of Sicilian people, like those of other populations living in Western countries, have greatly changed, and wild vegetable flavors are almost unknown to young people [75]. The elderly and those who still have strong links with the country follow a strictly Mediterranean-style diet instead. They know the best gathering seasons for the wild vegetables, and they are able to recognize and cook them according to established traditional practices [75]. In recent years, several studies on wild food plants have been carried out to preserve the traditional knowledge linked to their use in Sicily [47–49, 76–96].

In this study, we contribute to this purpose by carrying out an ethnobotanical survey of the wild plants still gathered and consumed as vegetables in Sicily. In several areas of the island, in fact, ancient traditions that allow us to understand the vegetable-based diets remain. The specific aims of this study are (1) to identify and record, through interviews with shepherds, farmers, and people who still have a close relationship with their environment, the edible taxa used as vegetables; (2) to compare the collected ethnobotanical data with the Italian and Mediterranean ethnobotanical international literature; and (3) to highlight possible new or unusual culinary plant uses.

## Methods

### Study area

**Fig. 1:**
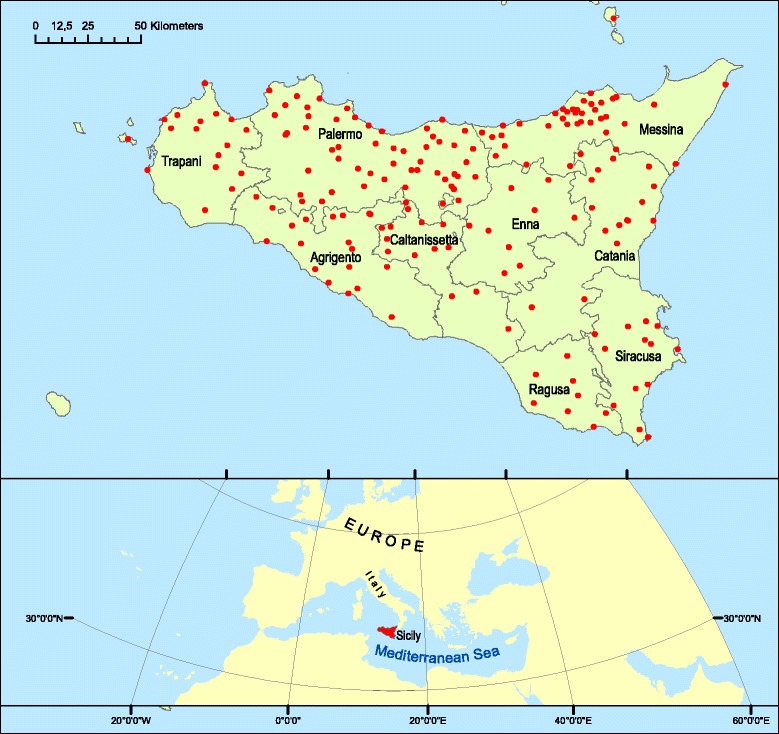
Map of the study area showing the centers and villages visited (red dots)

Sicily is the largest Italian island (Fig. 1), with an area of approximately 25,500 km^2^ and approximately 1000 km of coastline, rising from sea level to 3340 m (Mount Etna) [97]. The island has diverse geological characteristics, which have shaped different landforms. The territory is hilly in the central and southwestern parts (approximately 61.4%), mountainous, especially in the northern and eastern parts (24.5%), and 14.1% consists of alluvial plains [97].

According to Bazan et al. [98], Sicily is divided into 25 bioclimatic belts (thermotypes and ombrotypes) from lower thermomediterranean low semiarid to lower cryomediterranean upper hyperhumid. This great range of environmental conditions and its complex paleo-geographic and human history make the island one of the Mediterranean biodiversity hotspots [99]. The current vascular flora is composed of 3252 specific and subspecific taxa—native, adventive, and naturalized—arranged in 880 genera of 134 families. The richest ones are Asteraceae, with 371 specific and infraspecific taxa, followed by Poaceae (300), Fabaceae (295), Brassicaceae (141), Apiaceae (135), Caryophyllaceae (133), Lamiaceae (113), Rosaceae (94), Scrophulariaceae (82), Orchidaceae (82), Cyperaceae (71), Ranunculaceae (61), Chenopodiaceae (57), and Boraginaceae (53) [100]. Endemic species make up 15.44%, of which 9.90% are exclusive to Sicily, 3.69% are shared with southern Italy, and 1.85% are shared among a limited number of Mediterranean territories. The exotic composition of the flora includes 408 adventive and naturalized taxa (12.55%) [100]. Floristic richness is related to a high habitat diversity expressed in terms of vegetation types. Gianguzzi et al. [101] report 36 types of vegetation for Sicily, 16 of which are related to zonal vegetation (forests, shrublands, garrigues, grasslands communities, etc.), 11 are related to azonal vegetation (chasmophitic, riparian, psammophilous, etc.), and 9 are related to anthropogenic vegetation (arable lands and extensive herbaceous crops, vineyards, olive groves and dry cultivation mosaics, orchards, built-up areas, etc.). Traditional agricultural systems are widespread and are structured as highly diversified land mosaics, which are significant containers of biodiversity, including many wild food plants due to elevated diffuse naturalness [102].

### Data collection

**Fig. 2:**
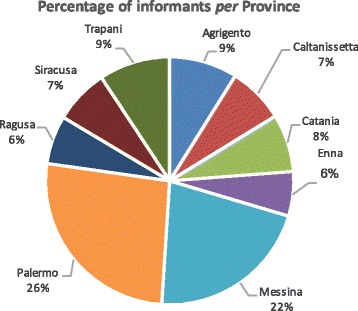
Distribution of informants in Sicilian provinces

**Fig. 3:**
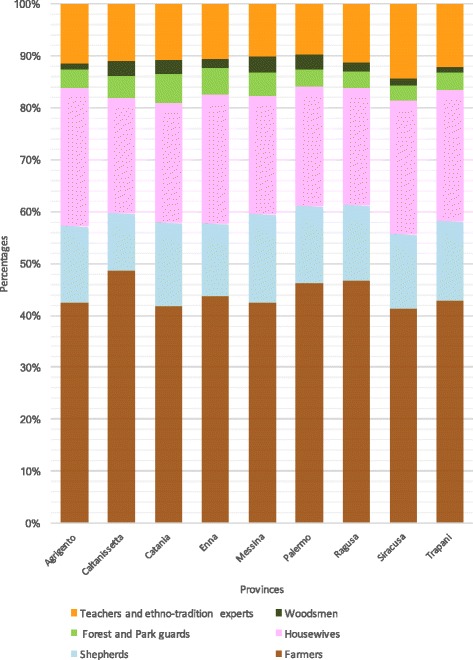
Categories of informants interviewed in Sicily

In the years 2005–2015, 187 towns and villages in Sicily were visited (Fig. 1), and randomly sampled people (54% men and 46% women) between the ages of 50 and 85 years (but primarily 65–75 years) for each town were interviewed after obtaining prior verbal informed consent (Fig. 2). The focus of the interviews (semi structured), which were frequently conducted either in Italian or Sicilian dialect, was their folk knowledge (name and use) of the wild vegetables that they still gather or that they ate in the past, especially during the war and post-war periods. The total number of interviewed people was 980: 433 farmers, 148 shepherds, 232 housewives, 38 forest and park guards, 23 woodsmen, and 106 teachers and ethno-tradition experts (Fig. 3). During or after the interview, the cited plants were usually collected together with the informants to confirm the correct identification of the plants. Sometimes, we gathered some specimens and showed them to the informants to confirm their edible uses. The Code of Ethics of the International Society of Ethnobiology was strictly followed [103].

The wild plant species mentioned by the informants were collected, when available, and identified according to *Flora d’Italia* [104] and stored at the Herbarium of the Museo Naturalistico F. Minà Palumbo (Castelbuono, Italy). Nomenclature follows the standards set by The Plant List database [105], in some cases Italian and Sicilian Checklists [100, 106, 107] and some recent publications [108, 109].

### Data analysis

In the present study, we have only considered data concerning the autochthonous plants (native species growing in their natural habitat), archaeophytes, and a few neophytes (introduced species that have been naturalized) traditionally gathered for food use. Following the classification for “food use” reported in Menenedez et al. [63], we have only analyzed the “vegetable” category (subcategories “processed vegetables” and “snacks”) and “flowers and stems” sucked for their sweet nectar (usually consumed to stimulate the appetite), and we excluded other uses (seeds, fruits, beverages, aromatics, seasonings, etc.). All the acquired data were processed, and some reports were drawn up in which for each plant there are (1) the scientific name and the family; (2) the life form sensu Raunkiær [110]; (3) the chorological element, distribution in Sicily, and habitat; (4) the Sicilian vernacular names (the two most common); (5) the edible parts following a modified version of the scheme proposed by Lentini and Venza [47]; (6) the traditional food use raw, cooked, or both; and (7) the estimated frequency of citations for each taxon (see Table 1).

**Table 1:** The list of wild vegetable plants used in the study area

	Taxa	Family	Life form	Chorotype	Habitat and distribution frequency	Vernacular names	Edible parts^a^	Food use^b^	Frequency of citations^c^
*	*Agave americana* L.	Agavaceae	P caesp	C-America	Uncultivated land, road edges both cultivated and spontaneous—C	*Zabbara, Zamara*	t-s	Co	R
	*Alliaria petiolata* (M. Bieb.) Cavara & Grande	Brassicaceae	H scap	Paleotemp.	Nitrophilous woods—C	*Agghialòra, Pedi d’asinu*	bu, le	Ra/Co	R
	*Allium ampeloprasum* L.	Amaryllidaceae	G bulb	Eurimedit.	Dry uncultivated land, edges of gardens—C	*Purrièttu, Puorru sarvaggiu*	bu, le, t-s	Ra/Co	VVC
*	*Allium nigrum* L.	Amaryllidaceae	G bulb	Stenomedit.	Fields, vineyards and olive-groves—C	*Agghiu d’i siminati, Porra*	bu	Ra/Co	VC
*	*Allium pendulinum* Ten.	Amaryllidaceae	G bulb	W-Stenomedit.	Woods, wet and shady ground—C	*Agghiu sarvaggiu*	le	Ra/Co	VC
	*Allium roseum* L.	Amaryllidaceae	G bulb	Stenomedit.	Garigue, dry meadows—VC	*Agghiu sarvaggiu, Porru*	bu	Ra/Co	C
	*Allium subhirsutum* L.	Amaryllidaceae	G bulb	Stenomedit.	Dry meadows, uncultivaded ground, and garigue—VC	*Agghiu sarvaggiu*	bu	Ra/Co	C
	*Allium triquetrum* L.	Amaryllidaceae	G bulb	W-Stenomedit.	Shady ground—C	*Agliotta, Porrua*	bu, le	Ra/Co	C
	*Allium ursinum* subsp. *ucrainicum* Kleopow & Oxner [100, 106]	Amaryllidaceae	G bulb	Eurasiat.	Beech-woods—NC	*Agghiu ursinu, Cipudda di serpi*	bu, le	Ra/Co	R
	*Amaranthus retroflexus* L.	Amaranthaceae	T scap	America Trop.	Ruins, debris, a weed in summer crops in dry and soft ground—C	*Lippia*	t-s	Co	R
	*Ammi majus* L.	Apiaceae	T scap	Eurimedit.	Uncultivated land, ruins, hoed fields—C	*Ènniri, Sberra*	le	Ra/Co	R
	*Anacyclus clavatus* (Desf.) Pers.	Asteraceae	T scap	Stenomedit.	Dry meadows, uncultivated land—VC	*Panipanuzzu*	t-s	Co	R
	*Anthemis arvensis* L. subsp. *arvensis*	Asteraceae	T scap	Stenomedit.	Cereal fields, pastures and uncultivated land—VC	*Cacumidda fitenti, Calumidda sarvaggia*	le	Co	R
	*Apium graveolens* L.	Apiaceae	H scap	Paleotemp.	Cultivated and wet uncultivated land—NC	*Accia sarvaggia, Accia*	le, t-s	Ra/Co	C
	*Apium nodiflorum* (L.) Lag.	Apiaceae	H scap	Eurimedit.	Ditches, ponds—C	*Scavùni, Crisciùni*	le, t-s	Ra/Co	C
*	*Arabis collina* Ten.	Brassicaceae	H scap	Medit.-Mont.	Grazing lands, cliffs, walls—C	*Razzi sarvaggi*	t-s	Co	R
*	*Arabis hirsuta* (L.) Scop.	Brassicaceae	H bienn	Europ.	Dry meadows, bushes, grazing lands, cliffs, road edges, walls—C	*Razzi*	t-s	Co	R
*	*Arabis turrita* L.	Brassicaceae	H bienn	S-Europ.-Sudsib.	Grazing land, deciduous, stony slopes and cliffs—R	*Mazarèddra duci, Cavulèdda*	le, t-s	Co	R
	*Arctium minus* (Hill) Bernh.	Asteraceae	H bienn	Eurimedit.	Uncultivated land, hedges, road edges, banks—NC	*Bardana*	le, t-s	Co	R
	*Asparagus acutifolius* L.	Asparagaceae	NP	Stenomedit.	Scrubland, holm oak, hedges Scrubland, holm oak, hedges—VC	*Spàracë di rizzògna, Sparacògna*	t-s	Ra/Co	VVC
	*Asparagus albus* L.	Asparagaceae	NP	W-Stenomedit.	Dry slopes, particularly in clayey ground and limestone—VC	*Sparaciu jancu, Spàraciu spinosu*	t-s	Ra/Co	VC
	*Asparagus aphyllus* L.	Asparagaceae	Ch frut	S-Stenomedit.	Dry and sunny slopes, hedges—VC	*Spàraciu nìuru*	t-s	Ra/Co	C
	*Asparagus horridus* L.	Asparagaceae	NP	S-Stenomedit.	Walls, hedges, garigue—NC	*Spàriciu marinu, Sparacògna sarvaggia*	t-s	Ra/Co	C
	*Asparagus officinalis* L.	Asparagaceae	G rhiz	Eurimedit.	Meadows and marshes—NC	*Sparaciu manzu, Sparaciu ‘mpiriali*	t-s	Ra/Co	C
	*Asphodeline lutea* (L.) Rchb.	Xanthorrhoeaceae	G rhiz	E-Eurimedit.	Dry meadows—VC	*Garùfi, Puddicìnu*	t-s	Co	C
	*Asphodelus ramosus* L. subsp. *ramosus* var. *ramosus* [100]	Xanthorrhoeaceae	G rhiz	Stenomedit.	Uncultivated dry ground, meadows—VC	*Purrazzu, Arvùzzi ramùsi*	ro	Co	R
*	*Asphodelus ramosus* subsp. *ramosus* var. *africanus* Z. Díaz & Valdés [100]	Xanthorrhoeaceae	G rhiz	Stenomedit.	Uncultivated clayey land—VC	*Agghiu porru, Purrazzu*	ro	Co	R
	*Barbarea vulgaris* R. Br.	Brassicaceae	H scap	Cosmop.	Wet muds, brook’s banks—R	*Caulicèddi di crapa, Lassana*	t-s	Co	C
*	*Bellis annua* L.	Asteraceae	T scap	Stenomedit.	Meadows, uncultivated land—C	*Erva di primu xiuri, Jancuzzu*	b-r	Ra/Co	C
.	*Bellis perennis* L. var. *perennis* [100]	Asteraceae	H ros	Europ.-Caucas.	Uncultivated land, meadows, disturbed sinantropic localites—C	*Erva di primu xiuri, Jancuzzu*	b-r	Ra/Co	C
*	*Bellis perennis* var. *hybrida* (Ten.) Fiori [100]	Asteraceae	H ros	Europ.-Caucas.	Meadows—R	*Erva di primu xiuri, Jancuzzu*	b-r	Ra/Co	R
*	*Bellis perennis* var. *strobliana* Bég. [100]	Asteraceae	H ros	Endem. Sic.	Mountain meadows—R	*Erva di primu xiuri, Jancuzzu*	b-r	Ra/Co	R
*	*Bellis sylvestris* Cirillo	Asteraceae	H ros	Stenomedit.	Uncultivated land, pastures, olive-grove—C	*Primu xiuri di voscu*	b-r	Ra/Co	R
	*Beta vulgaris* L. subsp. *vulgaris*	Chenopodiaceae	H scap	Eurimedit.	Wild on the coasts and commonly cultivated—VC	*Giri, Salachi*	le	Co	VVC
	*Beta vulgaris* subsp. *maritima* (L.) Arcang. [100, 106]	Chenopodiaceae	H scap	Eurimedit.	Along the coasts—VC	*Giri, Zarchi*	le	Co	VVC
*	*Biscutella maritima* Ten.	Brassicaceae	T scap	Endem.	Uncultivated dry ground—VC	*Cassatèddi, Ucchialèddi di Santa Lucia*	t-s	Co	R
	*Borago officinalis* L.	Boraginaceae	T scap	Eurimedit.	Uncultivated land, ruins—VC	*Vurrania, Bburraina*	fl/infl, le, t-s	Co	VVC
	*Brassica fruticulosa* Cirillo	Brassicaceae	H scap	W-Stenomedit.	Uncultivated land, walls, debris—VC	*Caulicèddu, Qualicèddu*	b-r, fl/infl	Co	VC
	*Brassica incana* Ten.	Brassicaceae	Ch suffr	Subendem.	Limestone cliffs, generally near the sea—NC	*Amarèddi*	fl/infl, t-s	Ra/Co	R
	*Brassica nigra* (L.) W. D. J. Koch	Brassicaceae	T scap	Eurimedit.	Cereal fields, uncultivated land, threshing-floors—C	*Cavulicèddu niuru, Mazzarèdda amara*	b-r, fl/infl, t-s	Co	C
	*Brassica rapa* subsp. *campestris* (L.) A. R. Clapham	Brassicaceae	T scap	Medit.	Fields, uncultivated land, road edges—VC	*Sinapë, Qualazzi*	b-r, fl/infl, t-s	Co	VVC
*	*Brassica rupestris* Raf. subsp. *rupestris*	Brassicaceae	Ch suffr	Endem.	Limestone cliffs—NC	*Cavulazzu, Càulu di rocca*	fl/infl, t-s	Ra/Co	R
*	*Brassica rupestris* subsp. *hispida* Raimondo & Mazzola [100, 106]	Brassicaceae	Ch suffr	Endem. Sic.	Limestone cliffs—NC	*Càulu sarvaggiu*	le	Ra/Co	VR
	*Brassica tournefortii* Gouan	Brassicaceae	T scap	Saharo-Sind.	Uncultivated dry ground, particularly maritime sands—NC	*Musulùchi*	le, t-s	Ra/Co	R
	*Bunias erucago* L.	Brassicaceae	T scap	N-Eurimedit.	Ruins, uncultivated grassy ground, hoed cultivations—C	*Ciconia di vigna, Spinacia sarvaggia*	le	Co	C
*	*Cakile maritima* Scop.	Brassicaceae	T scap	Eurosib.	Pioneer on costal sands and salty ruins—VC	*Arùca marina, Arùcula di mari*	t-s	Co	R
	*Calendula arvensis* (Vaill.) L. subsp. *arvensis*	Asteraceae	T scap	SW-Stenomedit.	Uncultivated land, road edges, fields and vineyards—VC	*Ciuri aranciu, Margherita russa*	b-r, t-s	Co	R
	*Capparis spinosa* L. subsp. *spinosa* var. *spinosa*	Capparidaceae	NP	Medit.	Gypsum cliffs, calanque—VC	*Chiappara, Chiàpparu manzu*	fl-b, t-s, fr	Ra/Co	VC
*	*Capparis spinosa* subsp. *rupestris* var. *rupestris*	Capparidaceae	NP	Medit.	Limestone cliffs and stony ground—VC	*Ciapparèdda, Ciàppiru*	fl-b, fr	Ra/Co	VC
*	*Capparis spinosa* subsp. *spinosa* var. *canescens* Cosson	Capparidaceae	NP	Medit.-Turan.	Gypsum and sulphur cliffs, calanque—VC	*Chiàpparu sarbaggiu, Chiàppara*	fl-b, fr	Ra/Co	VC
	*Capsella bursa-pastoris* (L.) Medik.	Brassicaceae	H bienn	Eurimedit.	Uncultivated land—VC	*Bbursa di picuràru, Mastròzzu sarvaggiu*	le	Co	R
	*Cardamine hirsuta* L.	Brassicaceae	T scap	Endem.	Cultivations, uncultivated land, grassland—VC	*Aruculicèdda sarvaggia, Crisciuneddu d’i mura*	t-s	Co	R
	*Carduus argyroa* Biv.	Asteraceae	T scap	Endem.	Uncultivated land, pastures, roadsides—VC	*Cacasagna, Napordi d’acqua*	t-s	Co	R
	*Carduus corymbosus* Ten.	Asteraceae	T scap	Endem.	Uncultivated dry ground, debris, roadsides—NC	*Carduneddu sarvaggiu*	t-s	Co	R
	*Carduus pycnocephalus* L. subsp. *pycnocephalus*	Asteraceae	H bienn	Eurimedit.-Turan.	Uncultivated land, road edges—VC	*Scoddi*	le	Co	R
	*Carlina gummifera* (L.) Less.	Asteraceae	H ros	S-Stenomedit.	Garigue, dry meadows—VC	*Masticògna, Cacucciulìdda*	fl/infl	Ra/Co	R
*	*Carlina hispanica* subsp. *globosa* (Arcang.) Meusel & Kästner	Asteraceae	H scap	Stenomedit.	Dry and stony meadows—C	*Mazzacugghiuna, Mazzacani*	t-s	Co	R
*	*Carlina sicula* Ten.	Asteraceae	H scap	Endem. Sic.	Uncultivated land, dry meadows, roadsides—C	*Carlina siciliana, Panicàudu*	t-s	Co	R
*	*Carrichtera annua* (L.) DC.	Brassicaceae	T scap	Stenomedit.-Turan.	Uncultivated dry ground—R	*Mastruzzu sarvaggiu*	t-s	Co	R
	*Carthamus lanatus* L. subsp. *lanatus*	Asteraceae	H scap	Eurimedit.	Clay-limestone ground—VC	*Vavanazzi, Carduni ‘nfiliniatu o ri spina*	t-s	Ra	R
	*Carthamus pinnatus* Desf.	Asteraceae	H ros	SW-Eurimedit.	Uncultivated land, pastures, garigue—C	*Carduncèllu*	b-r	Co	R
	*Centaurea calcitrapa* L.	Asteraceae	H bienn	Eurimedit.	Uncultivated dry ground, vineyards, roadsides—VC	*Apròcchi ri picucara, Sciaccablisci*	b-r	Co	C
*	*Centaurea napifolia* L.	Asteraceae	T scap	SW-Stenomedit.	Fields, uncultivated land, pastures hedges—VC	*Lucia*	b-r	Co	C
	*Centaurea sicula* L.	Asteraceae	H bienn	SW-Stenomedit.	Uncultivated land, roadsides—C	*Appròcchiu, Buttùni d’oru*	b-r, le	Co	R
	*Centaurea solstitialis* subsp. *schowii* (DC.) Dostál	Asteraceae	H bienn	Subendem.	Uncultivated land, vineyards, roadsides—C	*Apròcchiu fimminedda, Gattaredda*	le	Co	R
	*Centranthus ruber* (L.) DC.	Valerianaceae	Ch suffr	Stenomedit.	Cliffs, old walls—VC	*Baddariàna russa, Giummu di carrabbinera*	le	Ra/Co	R
	*Cerinthe major* L. subsp. *major*	Boraginaceae	T scap	Stenomedit.	Uncultivated land, vineyards edges and olive-grove, roadsides—VC	*Sucameli, Vrischi di api*	le	Ra/Co	C
	*Chamaemelum fuscatum* (Brot.) Vasc.	Asteraceae	T scap	W-Medit.-Mont.	Meadows and uncultivated wet ground—C	*Cacumidda, Pan’i cavaddu*	t-s	Ra/Co	R
	*Chamaerops humilis* L.	Arecaceae	P scap	W-Stenomedit.	Limestone cliffs and slopes on garigue Coastal belt—VC	*Giummarra, Scupazzu*	t-s	Ra	R
	*Chenopodium album* L.	Chenopodiaceae	T scap	Europa E-Asia	Uncultivated ground, ruins, a weed of cultivations—VC	*Erva fitenti, Inìsca*	le, t-s	Co	R
	*Chondrilla juncea* L.	Asteraceae	H scap	S-Europ.-Sudsib.	Uncultivated land and dry meadows—VC	*Curi î suggi, Cutulidda*	le, t-s	Co	C
	*Cichorium intybus* L. var. *intybus* [100, 106]	Asteraceae	H scap	Paleotemp.	Roadsides, in uncultivated land and ruins, a weed also in gardens—VC	*Cicòria,Cicoira*	b-r, le	Ra/Co	VVC
*	*Cichorium intybus* var. *glabratum* (C. Presl) Fiori [100, 106]	Asteraceae	H scap	Medit.	Mountain grasslands—NC	*Cicòria,Cicoira*	b-r, le	Ra/Co	C
*	*Cichorium pumilum* Jacq.	Asteraceae	T scap	Stenomedit.	Ruins, uncultivated land—C	*Nirvia sarvaggia*	b-r, le	Ra/Co	C
	*Clematis vitalba* L.	Ranunculaceae	P lian	Europ.-Caucas.	Sub-Mediterranean deciduous woods, hedges—VC	*Liàra, Mutarva*	t-s	Co	C
	*Clinopodium nepeta* (L.) Kuntze subsp. *nepeta*	Lamiaceae	H scap	Orof. S-Europ.	Dry meadows, uncultivated land, walls—VC	*Nipitedda, Niputeddra*	le	Ra/Co	C
	*Crepis bursifolia* L.	Asteraceae	H scap	Subendem.	Uncultivated land, dry meadows—VC	*Ricuttedda, Rizzaredda*	b-r, le	Co	C
	*Crepis leontodontoides* All.	Asteraceae	H ros	W-Medit.-Mont.	Forests, bushes, glads—C	*Rizzaredda*	b-r	Co	C
	*Crepis neglecta* subsp. *corymbosa* (Ten.) Nyman	Asteraceae	T scap	Subendem.	Uncultivated land, vineyards, roadsides—R	*Radicchiedda*	b-r	Co	R
*	*Crepis sprengelii* Nicotra	Asteraceae	H ros	Endem. Sic.	Fields, meadows and hedges—R	*Radicchiedda siciliana*	b-r	Co	R
	*Crepis vesicaria* L. subsp. *vesicaria*	Asteraceae	T scap	Eurimedit.-Subatl.	Uncultivated land, vineyards, roadsides	*Cicoria missinìsa, Cicoria vessicaria*	b-r, le	Co	VC
*	*Crepis vesicaria* subsp. *bivonana* (Soldano & Conti) Giardina & Raimondo	Asteraceae	T scap	Endem. Sic.	Uncultivated land and roadsides—VC	*Cicòria vessicaria,Cicuriuni*	b-r	Co	C
	*Crepis vesicaria* subsp. *hyemalis* (Biv.) Babc.	Asteraceae	T scap	Endem. Sic.	Uncultivated land, vineyards, roadsides—C	*Luciazzi*	b-r, le	Co	VC
	*Crepis vesicaria* subsp. *taraxacifolia* (Thuill.) Thell.	Asteraceae	T scap	W-Medit.	Uncultivated land and roadsides—C	*Cicoria amara, Lattuchedda di lu Signuri*	b-r, le	Co	C
	*Crithmum maritimum* L.	Apiaceae	Ch suffr	Eurimedit.	Maritime cliffs and reefs—VC	*Erva di lu pitittu, Finocchiu marinu*	le, t-s	Ra	R
	*Cynara cardunculus* L. subsp. *cardunculus*	Asteraceae	H scap	Stenomedit.	Pastures, uncultivated land—VC	*Cardùn’i spini, Cacòcciuliddu spinusu*	fl/infl, t-s	Ra/Co	VC
	*Cyperus esculentus* L.	Cyperaceae	He	Subcosmop.	Cultivated in marshes on the coast—C	*Cabbasìsi di Trapani, Nziparèddu*	ro	Co	C
	*Daucus carota* L. subsp. *carota*	Apiaceae	H bienn	Paleotemp.	Uncultivated land, roadsides, dry meadows—VC	*Vastunàca sarvaggia, Pedi di gaddu*	b-r, le	Ra/Co	R
	*Daucus carota* subsp. *maximus* (Desf.) Ball	Apiaceae	H bienn	Medit.-Asia	Uncultivated land, roadsides, dry meadows—NC	*Cuda di gattu*	t-s	Ra/Co	R
*	*Descurainia sophia* (L.) Prantl	Brassicaceae	T scap	Paleotemp.	Uncultivated land, ruins, often near stables—R	*Làssinu di sceccu, Mazzarèddri*	t-s	Co	R
	*Dioscorea communis* (L.) Caddick & Wilkin	Dioscoraceae	G rad	Eurimedit.	Woods, glads, hedges—VC	*Spàraciu arrampicusu, Spàrac’i serpi*	t-s	Co	C
	*Diplotaxis erucoides* (L.) DC. var. *erucoides* [100]	Brassicaceae	T scap	W-Stenomedit.	Fallow and uncultivated land—VC	*Xiuri di morti, Ruca sarvaggia*	le, t-s	Co	VC
	*Diplotaxis harra* subsp. *crassifolia* (Raf.) Maire	Brassicaceae	Ch suffr	S-Stenomedit.	Gypsum cliffs—C	*Erva cavulàra, Cavulicèddi*	le, t-s	Co	C
	*Diplotaxis muralis* (L.) DC.	Brassicaceae	T scap	Eurimedit.-Subatl.	Uncultivated land, ruins, road edges—R	*Erva diàvula, Erva diaulìna*	le, t-s	Co	R
	*Diplotaxis tenuifolia* (L.) DC.	Brassicaceae	H scap	Subatlant.	Ruins, uncultivated dry sandy ground—VC	*Ruca, Arùca sarvaggia*	le	Co	VC
	*Echium italicum* L. subsp. *italicum*	Boraginaceae	H bienn	Medit.	Dry mountain meadows—VR	*Acchiàppa muschi, Lingua di voi*	le	Co	R
*	*Echium italicum* L. subsp. *siculum* (Lacaita) Greuter & Burdet	Boraginaceae	H bienn	Endem. Sic.	Uncultivated land and dry meadows—VC	*Acchiàppa muschi, lingua viperina*	le	Co	R
	*Echium plantagineum* L.	Boraginaceae	T scap	Eurimedit.	Uncultivated dry and sandy ground along the coast and roadsides—VC	*Lapazza, Lingua di voi*	le	Co	R
	*Eruca vesicaria* subsp. *sativa* (Mill.) Thell.	Brassicaceae	T scap	Eurimedit.-Turan.	Ruins, gardens—C	*Arùca, Arùca sarvaggia*	le, t-s	Ra/Co	C
	*Erucastrum virgatum* (J. & C. Presl) C. Presl	Brassicaceae	H scap	Subendem.	Ruins and uncultivated land, pastures—R	*Sinapi, Càvulu sarvaggiu*	le, t-s	Co	R
	*Eryngium campestre* L.	Apiaceae	H scap	Eurimedit.	Dry meadows on limestone—VC	*Panicauru, N’zalata du diavulu,*	le	Ra	R
	*Fedia graciliflora* Fisch. & C. A. Mey.	Valerianaceae	T scap	Stenomedit.	Uncultivated land, roadsides and in gardens—C	*Peri ciocca, Lattucheddra di San Giuseppi*	le	Ra/Co	C
	*Foeniculum vulgare* Mill. subsp. *vulgare*	Apiaceae	H scap	S-Eurimedit.	Dry uncultivated land—VC	*Finucchieddu sarbaggiu, Finucchieddu rizzu,*	le, t-s	Ra/Co	VVC
	*Galactites elegans* (All.) Soldano [100, 106]	Asteraceae	H bienn	Stenomedit.	Uncultivated land, ruins, roadsides—VC	*Spina janca, Cardunèddu fimminèdda*	t-s	Co	R
*	*Gladiolus communis* L. subsp. *byzantinus* (Mill.) A. P. Ham. [100, 106]	Iridaceae	G bulb	Stenomedit.	Cereal fields—C	*Spatuliddra*	st-j	Ra	R
*	*Gladiolus communis* L. subsp. *communis*	Iridaceae	G bulb	S-Europ.-Sudsib.	Dry meadows—C	*Spatuliddra*	st-j	Ra	R
	*Gladiolus italicus* Mill.	Iridaceae	G bulb	Eurimedit.	Cereal fields—VC	*Spatuliddra*	st-j	Ra	R
	*Glebionis coronaria* (L.) Spach	Asteraceae	T scap	Stenomedit.	Fields, vineyards, olive-grove, uncultivated land—VC	*Sciùri di maju, Ciuri di cacamaiu*	t-s	Co	R
	*Hedypnois cretica* (L.) Dum.-Cours.	Asteraceae	T scap	Stenomedit.	Uncultivated land garigue, dry meadows	*Erva cracchiola*	t-s	Co	R
	*Hedypnois rhagadioloides* (L.) F. W. Schmidt	Asteraceae	T scap	Stenomedit.	Uncultivated land garigue, dry meadows	*Erva cracchiola*	t-s	Co	R
	*Helminthotheca echioides* (L.) Holub	Asteraceae	T scap	Eurimedit.	Hedges, road sides, dry meadows, ruins—VC	*Spirèdda, Asparèdda*	le	Co	R
	*Himantoglossum robertianum* (Loisel.) P. Delforge	Orchidaceae	G bulb	Stenomedit.	Dry meadows, garigue and small bushes—VC	*Patatara, Gaddùzzi*	bu, ro	Co	R
	*Hirschfeldia incana* (L.) Lagr.-Foss.	Brassicaceae	H scap	Eurimedit.	Ruins, uncultivated land, along the roads—VC	*Làssimi, Mazzareddi*	fl/infl, le, t-s	Co	VC
	*Hyoseris radiata* L.	Asteraceae	T ros	Stenomedit.	Uncultivated grassy ground, walls, slopes, stony paths—VC	*Occhi di pirnici, Cicuriuni*	b-r	Co	VC
*	*Hyoseris scabra* L.	Asteraceae	T ros	Stenomedit.	Uncultivated dry ground, near the coast—NC	*Cicuriuni, Erba duci*	b-r	Co	C
	*Hypochaeris achyrophorus* L.	Asteraceae	T scap	Stenomedit.	Uncultivated land and dry meadows—VC	*Costa ri vecchia, Cicoria lingua di jatta*	b-r, le	Co	VC
	*Hypochaeris cretensis* (L.) Bory & Chaub.	Asteraceae	H scap	NE-Medit.-Mont.	Dry and stony slopes, mountain pastures—C	*Cìtula duci*	b-r, le	Co	C
	*Hypochaeris glabra* L.	Asteraceae	T scap	Eurimedit.	Uncultivated dry ground, pastures—C	*Costi vecchia*	b-r, le	Co	VC
	*Hypochaeris laevigata* (L.) Ces.	Asteraceae	H ros	SW-Medit.-Mont.	Cliffs, stony pastures—C	*Razza*	b-r, le	Co	VC
	*Hypochaeris radicata* L.	Asteraceae	H ros	Europ.-Caucas.	Sands, dry meadows, uncultivated land—C	*Cicoria furfuciata, Sgàrri*	b-r, le	Co	VC
*	*Iris tuberosa* L.	Iridaceae	G rhiz	N-Stenomedit.	Uncultivated land, hedges, and olive groves—VC	*Buttùni di jaddu, Castagnotto*	ro	Co	C
	*Isatis tinctoria* subsp. *canescens* (DC.) Arcang. [100]	Brassicaceae	H bienn	SE-Asia	Uncultivated land, along the roads—VC	*Cavulu di carammu, Guàdu*	fl/infl	Co	R
*	*Jacobaea erratica* (Bertol.) Fourr.	Asteraceae	H bienn	C-Europ.	Wet and shady localities—C	*Erva rapudda, Erva di San Giacumu*	le	Co	R
*	*Juncus acutus* L.	Juncaceae	H caesp	Eurimedit.	Wet salt sandy ground, embankments, clayey ground—VC	*Juncu, Junci di liari*	t-s	Co	R
*	*Kundmannia sicula* (L.) DC.	Apiaceae	H scap	Stenomedit.	Dry uncultivated land, pastures—C	*Pedi di nigli, Pitrusinu sarvaggiu*	le	Co	VR
	*Lactuca muralis* (L.) Gaertn.	Asteraceae	H scap	Europ.-Caucas.	Woods—C	*Cardedda di muru*	le	Ra/Co	C
	*Lactuca serriola* L.	Asteraceae	H bienn	S-Europ.-Sudsib.	Uncultivated land, vineyards, roadsides—VC	*Lattuca sarbaggia, Lattùca spinusa*	le	Ra/Co	C
	*Lactuca viminea* (L.) J. & C. Presl	Asteraceae	H bienn	Europ.-Caucas.	Dry and stony slopes—VC	*Lattughèdda du Signuri, Evva di scussuni*	le	Ra/Co	C
*	*Lamium flexuosum* Ten.	Lamiaceae	H scap	NW-Medit.-Mont.	Stony ground, wet cliffs, scrubland—R	*Nzinzili*	st-j	Ra	R
	*Lapsana communis* L.	Asteraceae	T scap	Paleotemp.	Broadleaf woods and fresh disturbed localities—C	*Lassani ruci, Erva pi li minni*	t-s	Ra/Co	R
	*Lathyrus annuus* L.	Fabaceae	T scap	Eurimedit.	Fields, pastures, uncultivated land—C	*Fasuolu sarvaggiu*	t-s	Co	R
	*Lathyrus sylvestris* L.	Fabaceae	H scand	Europ.-Caucas.	Dry meadows, hedges—C	*Cessavuoi, Fasòla sarvaggia*	fl/infl, t-s	Co	R
*	*Leontodon cichoraceus* (Ten.) Sanguin.	Asteraceae	H ros	Orof. SE-Europ.	Uncultivated dry ground, pastures, hedges—R	*Cicuriedda*	b-r	Co	VC
*	*Leontodon intermedius* Huter, Porta & Rigo	Asteraceae	H ros	Endem.	Limestone cliffs—C	*Cicuriedda*	b-r	Co	C
*	*Leontodon muelleri* (Sch. Bip.) Fiori	Asteraceae	T scap	S-Stenomedit.	Pastures and uncultivated wet ground—R	*Occhiu di pinnici*	b-r	Co	C
*	*Leontodon siculus* (Guss.) Nyman	Asteraceae	H ros	Endem.	Beech and Turkey oak woods—R	*Lattughedda di muntagna*	b-r	Co	C
	*Leontodon tuberosus* L.	Asteraceae	H ros	Stenomedit.	Dry meadows, olive-grove, glades in scrublands—VC	*Occhiu di pinnici, Lattughedda*	b-r	Co	C
	*Leopoldia comosa* (L.) Parl.	Hyacinthaceae	G bulb	Eurimedit.	Fields, uncultivated dry ground—VC	*Cipuddazza, Agghiòru niuru,*	bu	Co	R
*	*Lepidium draba* L.	Brassicaceae	G rhiz	Giamaica	Uncultivated land along the roads, ruins—VC	*Aruchèdda, Erva pipirìna*	t-s	Co	R
	*Lepidium graminifolium* L.	Brassicaceae	H scap	Eurimedit.	Road sides, ruins—VC	*Mastrùzzu sarvaggiu*	t-s	Co	R
*	*Lepidium latifolium* L.	Brassicaceae	H scap	Subendem.	Uncultivated dry barren ground—R	*Erva pipirìtu, Erva mustarda*	t-s	Co	R
*	*Lobularia maritima* (L.) Desv. subsp. *maritima*	Brassicaceae	H scap	Stenomedit.	Uncultivated dry ground, cliffs, walls—VC	*Qualidduzzu profumatu, Ciùri bbiàncu*	t-s	Co	R
	*Lycium europaeum* L.	Solanaceae	NP	Eurimedit.	Cultivated for hedges and grown wild along interpoderal roads—C	*Spinasanta, Tammuscèddu*	t-s	Co	C
	*Malva cretica* Cav.	Malvaceae	T scap	Stenomedit.	Dry uncultivated land—C	*Marva*	le	Co	C
*	*Malva multiflora* (Cav.) Soldano, Banfi & Galasso	Malvaceae	T scap	Stenomedit.	Dry uncultivated land, fields, ruins—VC	*Marva, Marvùni*	le	Co	C
	*Malva nicaeensis* All.	Malvaceae	T scap	Stenomedit.	Dry uncultivated land, paths, pastures—C	*Marva, Marba*	le	Ra/Co	C
	*Malva parviflora* L.	Malvaceae	T scap	Stenomedit.	Uncultivated land near the houses—C	*Panicèdda, Pani-panùzzi*	le	Co	C
	*Malva sylvestris* L. subsp. *sylvestris* [100, 106]	Malvaceae	H scap	Eurosib.	Wasteland piles of debris and rubbish—VC	*Marva, Mavvàscu*	le	Co	C
*	*Malva sylvestris* subsp. *ambigua* (Guss.) Thell. [100]	Malvaceae	H scap	Eurosib.	Wasteland piles of debris and rubbish—C	*Marva, Mavvàscu*	le	Co	C
*	*Malva trimestris* (L.) Salisb. [100]	Malvaceae	T scap	Stenomedit.	Fields, uncultivated land and pastures—VC	*Marva, Marvùni*	le	Co	C
	*Moricandia arvensis* (L.) DC.	Brassicaceae	T scap	S-Stenomedit.	Ruins, uncultivated land, often along the railways—VC	*Càvulu sarvaggiu, Garòfalu sarvaggiu*	le, t-s	Co	R
*	*Narcissus tazetta* L. subsp. *tazetta*	Amaryllidaceae	G bulb	Stenomedit.	Meadows—VC	*Narcìsu, Agghi porri*	fl/infl	Ra/Co	VR
	*Nasturtium officinale* R. Br.	Brassicaceae	H scap	Cosmop.	Still and running waters, banks—VC	*Crisciuni, Scavùni*	le, t-s	Ra/Co	C
	*Notobasis syriaca* (L.) Cass.	Asteraceae	T scap	Stenomedit.	Fields, uncultivated land, dry meadows, roadsides—VC	*Piscialàsinu, Lamànna*	t-s	Ra/Co	R
*	*Onopordum horridum* Viv.	Asteraceae	H bienn	NE-Medit.-Mont.	Uncultivated land, rubbish dump, covili—C	*Napòrdu*	b-r	Co	VC
	*Onopordum illyricum* L. subsp. *illyricum*	Asteraceae	H bienn	Stenomedit.	Uncultivated land, debris, near the stables—VC	*Napruddri, Munaceddu*	b-r	Co	VC
*	*Opuntia ficus-indica* (L.) Mill.	Cactaceae	P succ	America Trop.	Dry localities and cliffs—VC	*Ficudinia, Fikupali*	fr	Co	R
	*Oxalis pes-caprae* L.	Oxalidaceae	G bulb	S-Africa	Uncultivated land, gardens, fields—VC	*Cannacitula, auriduci*	bu, le, st-j	Ra/Co	C
	*Papaver rhoeas* L. var. *rhoeas* [100]	Papaveraceae	T scap	E-Medit.-Mont.	A weed of cereal cultivation, and ruderal	*Paparina russa, Paparinazzu*	le	Ra/Co	C
*	*Papaver rhoeas* var. *himerense* Raimondo & Spadaro [100]	Papaveraceae	T scap	Endem. Sic.	Nitrophylous open sites—RR	*Papaviru rosa*	le, t-s	Ra/Co	R
	*Papaver somniferum* subsp. *setigerum* (DC.) Arcang.	Papaveraceae	T scap	W-Medit.-Mont.	Pastures, walls and cultivations—NC	*Paparina manza*	le	Ra/Co	VR
	*Picris hieracioides* subsp. *spinulosa* (Guss.) Arcang.	Asteraceae	H scap	Eurosib.	Uncultivated land, roadsides—VC	*Spireddra*	le	Co	R
*	*Plantago afra* L.	Plantaginaceae	T scap	Stenomedit.	Uncultivated dry ground, pastures—VC	*Erva d’i purci, Pisillìna*	b-r	Co	R
	*Plantago coronopus* L. subsp. *coronopus*	Plantaginaceae	T scap	Eurimedit.	Uncultivated dry ground, near the sea, salt meadows, reefs—C	*Cornopiu, Erva di stidda*	b-r	Co	C
	*Plantago lagopus* L.	Plantaginaceae	T scap	Stenomedit.	Dry meadows, uncultivated land—C	*Cutidduzzi, Cuda di gatta*	b-r	Co	R
	*Plantago lanceolata* L. var. *lanceolata* [100]	Plantaginaceae	H ros	Eurasiat.	Uncultivated land, roadsides, fields, vineyards, generally sinantrophic—C	*Lanzafina, Centunèrvi strittu*	b-r	Co	C
	*Plantago major* L. subsp. *major*	Plantaginaceae	H ros	Eurasiat.	Moist mountain localities drying in Spring—C	*Centunèrvi, Pampina larga*	b-r	Co	R
	*Plantago serraria* L.	Plantaginaceae	H ros	Stenomedit.	Uncultivated dry ground mainly on the coastland—C	*Tuonachi, Chirchi di parrini*	b-r	Co	C
	*Portulaca oleracea* L. subsp. *oleracea*	Portulacaceae	T scap	Subcosmop.	Fields, gardens, uncultivated ground—VC	*Purciddana, Pucciddana*	le, t-s	Ra/Co	VC
	*Primula vulgaris* Huds.	Primulaceae	H ros	Europ.-Caucas.	Broadleaf woods—C	*Conterba siciliana, Sciuri a scocca*	b-r	Ra/Co	R
	*Raphanus raphanistrum* L. subsp. *raphanistrum*	Brassicaceae	T scap	Eurimedit.	Ruins, gardens, often also a weed of cultivations—VC	*Razza ruci, Lapistra*	le, t-s	Co	VC
	*Raphanus raphanistrum* subsp. *landra* (DC.) Bonnier & Layens	Brassicaceae	T scap	Eurimedit.	Ruins and fields—VC	*Mazzaredda, Razza*	le, t-s	Co	VC
*	*Raphanus raphanistrum* subsp.* maritimus* (Sm.) Thell. [100]	Brassicaceae	T scap	Eurimedit.	Ruins and fields near the sea—C	*Ràfanu sarvaggiu, Aràzzu*	le, t-s	Co	C
	*Rapistrum rugosum* subsp. *orientale* (L.) Arcang. [100, 106]	Brassicaceae	T scap	Eurimedit.	Uncultivated dry land, grazing, road edges—C	*Sinàpa spagnola*	le, t-s	Co	C
	*Reichardia picroides* (L.) Roth	Asteraceae	H scap	Stenomedit.	Maritime cliffs, uncultivated dry ground, walls, roadsides—VC	*Caccialiepru, Curcita*	b-r	Co	VVC
	*Rhagadiolus stellatus* (L.) Gaertn.	Asteraceae	T scap	Eurimedit.	Uncultivated land, fields, dry meadows—C	*Raricchiu sarvaggiu*	b-r, le	Co	C
	*Ridolfia segetum* Moris	Apiaceae	T scap	Stenomedit.	Cereal fields—VC	*Finocchiu anitu, Finucciàzzu*	t-s	Ra	R
*	*Rorippa sylvestris* (L.) Besser	Brassicaceae	H scap	Eurasiat.	Muds, uncultivated wet ground—VR	*Arùca sarvaggia picciridda*	le	Co	VR
	*Rosa canina* L.	Rosaceae	NP	Paleotemp.	Degraded scrubland, bushes, and hedges—VC	*Giarrauta, Rosa sarvaggia*	fl/infl	Ro	R
	*Rosa sempervirens* L.	Rosaceae	NP	W-Medit.-Mont.	Thermo-Meso-Mediterranean woods and scrublands—C	*Rusidda spinusa, Rusidda di San Giuvanni*	fl/infl	Ro	R
	*Rubus ulmifolius* Schott	Rosaceae	NP	Eurimedit.	Hedges, uncultivated land, coppice—VC	*Amurèdda, Rivèttu*	t-s	Ra/Co	VR
	*Rumex acetosa* L.	Polygonaceae	H scap	Circumbor.	Manured and mown meadows—R	*Acitàzzu, Aureddùci*	t-s	Co	R
	*Rumex bucephalophorus* L. subsp. *bucephalophorus*	Polygonaceae	T scap	Eurimedit.-Macaron.	Uncultivated dry ground mainly on the coastland—VC	*Acitusèdda, Agru-duci cu’ fogghi picciriddi*	t-s	Co	R
	*Rumex crispus* L.	Polygonaceae	H scap	Subcosmop.	Uncultivated and cultivated ground, ruins—C	*Aùru acìtu, Lapàzzu*	t-s	Co	VR
	*Rumex intermedius* DC.	Polygonaceae	H scap	NW-Stenomedit.	Uncultivated ground—R	*Acitàzzu*	t-s	Co	R
	*Rumex pulcher* L. subsp. *pulcher*	Polygonaceae	H scap	Eurimedit.	Uncultivated land, ruins, meadows and semi-humid ground—VC	*Lapàzza, Lapazzèddu rizzu*	t-s	Co	R
	*Rumex scutatus* L.	Polygonaceae	H scap	S-Europ.-Sudsib.	Limestone stony and uncultivated land—VC	*Acìtula di sciara, Citulìdda*	le, st-j	Ra/Co	R
	*Rumex thyrsoides* Desf.	Polygonaceae	H scap	W-Stenomedit.	Dry uncultivated ground—VC	*Acìtura*	t-s	Co	R
	*Ruscus aculeatus* L.	Ruscaceae	Ch frut	Eurimedit.	Thermophilous Quercus woods—C	*Spinasurci, Scuparìni*	t-s	Ra/Co	VVC
	*Ruscus hypophyllum* L.	Ruscaceae	Ch frut	Eurimedit.	Broadleaf woods, particularly *Quercus ilex* woods—R	*Sparaci trona, Erva di trònu*	t-s	Ra/Co	C
.	*Salvia officinalis* L.	Lamiaceae	Ch suffr	N-Medit.-Mont.	Only rarely naturalized, and always in disturbed habitat—R	*Sarvia*	le	Ra/Co	C
	*Sambucus nigra* L.	Caprifoliaceae	P caesp	Europ.-Caucas.	Wet woods, glades, hedges—NC	*Sammùccu, Savùcu*	fl/infl	Co	R
	*Sanguisorba minor* Scop. subsp. *minor*	Rosaceae	H scap	Paleotemp.	Dry meadows, garigue, uncoltivated ground—NC	*Pampinèdda di campagna, Pimpinedda*	le	Co	VR
	*Scolymus grandiflorus* Desf.	Asteraceae	H scap	SW-Eurimedit.	Uncultivated land, road edges—VC	*Scòddi, Zammuri di campagna*	t-s	Ra/Co	VC
	*Scolymus hispanicus* L.	Asteraceae	H bienn	Eurimedit.	Uncultivated dry and sandy ground—VC	*Spina bianca, Scoddu*	t-s	Ra/Co	C
	*Scolymus maculatus* L.	Asteraceae	T scap	S-Stenomedit.	Uncultivated clayey ground—VC	*Scoddu, Scuoddo*	t-s	Ra/Co	C
	*Scorzonera cana* (C. A. Mey.) Griseb.	Asteraceae	H scap	S-Europ.-Sudsib.	Clayey and marly ground—C	*Benedìciti*	le	Co	R
	*Scorzonera laciniata* L.	Asteraceae	H bienn	Paleotemp.	Uncultivated land, vineyards, dry slopes—NC	*Erva di gnàgnaru pilusa, Scursunèra*	le, t-s	Ra/Co	R
*	*Scorzonera laciniata* subsp. *decumbens* (Guss.) Greuter [106]	Asteraceae	H bienn	Medit.	Vineyards, cultivation edges, ruins—NC	*Latti di lepri*	le, t-s	Co	R
	*Scorzonera undulata* subsp. *deliciosa* (Guss.) Maire	Asteraceae	G bulb	SW-Stenomedit.	Uncultivated dry ground—C	*Scursunèra*	b-r, ro	Ra/Co	R
	*Senecio vulgaris* L.	Asteraceae	T scap	Eurimedit.	Uncultivated land near houses and a weed in fields—VC	*Erva di li cardìddi, Mancialèbbri*	le	Co	R
	*Silene vulgaris* (Moench) Garcke subsp. *vulgaris*	Caryophyllaceae	H scap	Paleotemp.	Uncultivated ground, meadows, scree—C	*Aricchi i liepru, Erba du priricaturi*	t-s	Ra/Co	VC
*	*Silene vulgaris* subsp. *commutata* (Guss.) Hayek	Caryophyllaceae	H scap	Orof. SE-Europ.	Meadows among cliffs—R	*Aricchi i liepru, Cannatèdda*	t-s	Ra/Co	C
*	*Silene vulgaris* subsp. *tenoreana* (Colla) Soldano & F. Conti [100, 106]	Caryophyllaceae	H scap	Steno - Medit –Orient	Dune, reefs, and dry localities near the sea—VC	*Calicèdda di muru, Campanèdda*	t-s	Ra/Co	C
	*Silybum marianum* (L.) Gaertn.	Asteraceae	H bienn	Eurimedit.-Turan.	Ruins, hedges, roadsides—VC	*Carduggiu, Cardu marianu*	b-r	Co	VC
	*Sinapis alba* L. subsp. *alba*	Brassicaceae	T scap	E-Medit.	Cereal fields, uncultivated land and ruins—VC	*Làssani, Mazzarèddu*	fl/infl, t-s	Co	R
*	*Sinapis alba* L. subsp*. dissecta* (Lag.) Bonnier	Brassicaceae	T scap	E-Medit.-Mont.	Cereal fields, uncultivated land and ruins—NC	*Sinacciòlu di linu*	fl/infl, t-s	Co	R
	*Sinapis arvensis* L.	Brassicaceae	T scap	Stenomedit.	Cereal fields, uncultivated land, ruins—VC	*Alàssani, Sinàpa sarvaggia*	fl/infl, t-s	Co	R
*	*Sinapis pubescens* L.	Brassicaceae	Ch suffr	SW-Stenomedit.	Uncultivated dry ground, cliffs—VC	*Sinacciòla, Sinàpa fimminedda*	fl/infl, t-s	Co	R
	*Sisymbrium irio* L.	Brassicaceae	T scap	Paleotemp.	Uncultivated land, ruins, gardens—VC	*Approcchiu, Pisciacani*	le	Ra/Co	R
	*Sisymbrium officinale* (L.) Scop.	Brassicaceae	T scap	Paleotemp.	Antropophilous on debris and road sides—VC	*Làssinu di sceccu, Mazzarèddri*	fl/infl	Ra/Co	R
	*Smilax aspera* L.	Smilacaceae	NP	Subtrop.	Evergreen scrubland, holm oak—VC	*Gratta culu, Stràzzacausi*	t-s	Co	R
	*Smyrnium olusatrum* L.	Apiaceae	H bienn	Eurimedit.-Subatl.	Wet and shady uncultivated land, hedges, ruins and debris—VC	*Làccia sarvaggia, Lisciànnaru*	t-s	Ra/Co	VR
	*Smyrnium perfoliatum* L.	Apiaceae	H bienn	Eurimedit.	Coppice and uncultivated shady ground—C	*Lisciandrèddu*	t-s	Ra/Co	VR
	*Smyrnium rotundifolium* Mill.	Apiaceae	H bienn	S-Stenomedit.	Dry and sunny uncultivated land—C	*Casese, Casesi*	t-s	Ra/Co	VR
	*Solanum americanum* Mill*.*	Solanaceae	T scap	Cosmopol.	Fields, uncultivated land, ruins—VC	*Amareddri, Pumarureddi niuri*	le, t-s	Co	R
	*Sonchus asper* (L.) Hill subsp. *asper*	Asteraceae	T scap	Eurasiat.	Hoed fields, gardens, vineyards—C	*Cardedda spinusa, Cardedda di scecchi*	b-r, le	Co	VVC
	*Sonchus asper* subsp. *glaucescens* (Jord.) Ball	Asteraceae	T scap	Eurasiat.	Uncultivated land mainly near the sea—R	*Cardiddazza, Cardinnastra*	b-r, le	Co	C
	*Sonchus oleraceus* L.	Asteraceae	T scap	Eurasiat.	Fields and abandoned fields—VC	*Cardedda bianca, Cardedda fimminina*	b-r, le	Co	VVC
	*Sonchus tenerrimus* L.	Asteraceae	T scap	Stenomedit.	Cliffs, fields, uncultivated land, urbn habitat—VC	*Cardedda di muru, Cardèdda scucìvola*	b-r, le	Co	VVC
	*Stellaria media* subsp. *cupaniana* (Jord. & Fourr.) Nyman	Caryophyllaceae	T scap	Medit.	Antropogen vegetation—VC	*Centocchiu*	le, t-s	Co	R
	*Stellaria media* (L.) Vill. subsp. *media*	Caryophyllaceae	T rept	Cosmopol.	Ruderal and a weed, human sites, gardens—NC	*Centocchiu*	t-s	Co	C
*	*Sulla coronaria* (L.) Medik.	Fabaceae	H scap	W-Stenomedit.	Clayey ground—C	*Sudda, Suddra*	t-s	Ra/Co	C
	*Taraxacum campylodes* G.E.Haglund	Asteraceae	H ros	Circumbor.	Hill and mountain meadows—NC	*Tarassacu, Denti di liuni*	b-r	Co	C
*	*Taraxacum caramanicae* Lojac.	Asteraceae	H ros	Endem. Sic.	Open fields, disturbed habitat—NC	*Tarassacu, Denti di liuni*	b-r	Co	C
*	*Taraxacum garbarianum* Peruzzi, Aquaro, Caparelli & Raimondo [100]	Asteraceae	H scap	Endem. Sic.	Mountain open pastures—R	*Tarassacu, Denti di liuni*	b-r	Co	R
*	*Taraxacum gasparrinii* Lojac.	Asteraceae	H ros	W-Eurimedit.	Woods—C	*Taràssacu*	b-r	Co	R
*	*Taraxacum minimum* (Guss.) N. Terracc.	Asteraceae	H ros	Medit.	Mountain open pastures—NC	*Cuddu cudduzzu, Cicòria sarvaggia*	b-r	Co	R
	*Taraxacum obovatum* (Willd.) DC.	Asteraceae	H ros	W-Medit.-Mont.	Meadows, road edges, disturbed habitat—C	*Erba di pirnici*	b-r	Co	R
*	*Taraxacum siculum* Soest	Asteraceae	H ros	Endem.	Wet localities with stagnant water—VR	*Denti di liuni sicilianu*	b-r	Co	VR
*	*Teucrium fruticans* L.	Lamiaceae	NP	W-Stenomedit.	Limestone cliffs near the sea—VC	*Alivedda, Caca aucèddi*	b-r	Co	R
	*Thlaspi perfoliatum* L.	Brassicaceae	T scap	Paleotemp.	Mountain grasslands—NC	*Talaspiu*	t-s	Co	R
*	*Tolpis umbellata* Bertol.	Asteraceae	T scap	Stenomedit.	Uncultivated land, dry meadows—C	*Scalurèdda*	b-r	Co	R
*	*Tolpis virgata (*Desf.) Bertol. subsp. *grandiflora* (Ten.)	Asteraceae	T scap	Endem.	Uncultivated land, dry meadows—NC	*Scalurèdda, Erba janca*	b-r	Co	R
*	*Tolpis virgata* (Desf.) Bertol. subsp. *virgata*	Asteraceae	T scap	Stenomedit.	Uncultivated land and dry meadows—NC	*Scalurèdda, Lattuchedda*	b-r	Co	R
	*Tordylium apulum* L.	Apiaceae	T scap	Stenomedit.	Dry meadows, cultivated and uncultivated land—VC	*Spiccialiccia, Tammuridduzzi picciriddi*	t-s	Ra	VR
	*Tragopogon crocifolius* subsp. *nebrodensis* (Guss.) Raimondo	Asteraceae	T scap	Endem. Sic.	Uncultivated land, dry meadows, roadsides—R	*Barbabècchi, Latti d’aceddu*	le, t-s	Co	R
	*Tragopogon porrifolius* L. subsp. *porrifolius*	Asteraceae	H bienn	Eurimedit.	Mountain pastures—VR	*Latti d’aceddi, Barbabecchi*	le, t-s	Co	R
	*Tragopogon porrifolius* subsp. *australis* (Jord.) Nyman	Asteraceae	H bienn	Medit.	Uncultivated land, dry meadows, roadsides—NC	*Erba di gnagnaru pilusa, Varva di beccu*	le	Ra/Co	R
*	*Tragopogon porrifolius* subsp. *cupanii* (DC.) I. Richardson	Asteraceae	H bienn	Endem.	Dry meadows, uncultivated land, roadsides and field edges—NC	*Varba di vecchiu*	le, t-s	Co	R
*	*Umbilicus horizontalis* (Guss.) DC.	Crassulaceae	H rhiz	Stenomedit.	Wet and shady cliffs, old walls—VC	*Paracqua, Aricchia di vecchia,*	le	Ra	R
*	*Umbilicus rupestris* (Salisb.) Dandy	Crassulaceae	G rhiz	Stenomedit.-Atl	Wet and shady cliffs, old walls—VC	*Pampina di uricchia, Uriccieddi*	le	Ra	R
	*Urospermum dalechampii* (L.) F. W. Schmidt	Asteraceae	H scap	Eurimedit.	Dry meadows, uncultivated land, roadsides—VC	*Cicoria sarvaggia, Cuosti i porci*	b-r, le	Co	VC
	*Urospermum picroides* (L.) F. W. Schmidt	Asteraceae	T scap	Eurimedit.	Uncultivated land, roadsides, olive-grove, vineyards—VC	*Cardiddazza spinusa*	b-r, le	Co	VC
	*Urtica dioica* L.	Urticaceae	H scap	Subcosmop.	Nitrophilous habitat, also in wood clearings and riverbeds—C	*Ardìcula fimminedda, Lardìca sarvaggia*	le	Co	C
	*Urtica membranacea* Poir.	Urticaceae	T scap	S-Stenomedit.	Ruins and nitrophilous habitat—VC	*Addrìcula, Ziculièdda*	le	Co	C
	*Urtica pilulifera* L.	Urticaceae	T scap	S-Stenomedit.	Ruins and nitrophilous habitat—VC	*Ardicula masculina*	le	Co	R
	*Urtica urens* L.	Urticaceae	T scap	Subcosmop.	In disturbed habitat, nitrophilous and often urophilous species—C	*Ardiculèdda fimminedda, Ddìcula*	le	Co	C
	*Valerianella eriocarpa* Desv.	Valerianaceae	T scap	Stenomedit.	A weed to sown lands, uncultivated land, pastures—VC	*Gaddinedda, Per’i ciocca*	le, t-s	Ra/Co	R
	*Valerianella locusta* (L.) Laterr.	Valerianaceae	T scap	Eurimedit.	Acid meadows—NC	*Gaddinedda, Spezzaquartàri*	le, t-s	Ra/Co	R
	*Veronica anagallis-aquatica* L. var. *anagallis-aquatica* [100]	Scrophulariaceae	H scap	Cosmopol.	Ditches, banks—VC	*Crisciunèddu, Erva di tràcina*	le	Ra	R
*	*Xanthium strumarium* L. subsp. *strumarium*	Asteraceae	T scap	America	Ruins, debris, uncultivated dry ground—VC	*Aggruppa cudi, Bardana minuri*	b-r	Co	R
*	*Xanthium orientale* subsp. *italicum* (Moretti) Greuter	Asteraceae	T scap	N-Eurimedit.	Uncultivated land, ruins near the sea—VC	*Aggruppa cudi, Bardana minuri*	b-r	Co	R

Asterisk indicates taxa used only in Sicily as vegetable

*Ch frut* fruticose chamaephytes, *Ch suffr* suffruticose chamaephytes, *G bulb* bulbous geophytes, *G rad* root-budding geophyte, *G rhiz* rhizome-geophytes, *H bienn* biennial hemicryptophytes, *H caesp* caespitose hemicryptophytes, *H rhiz* rhizomatous hemicryptophytes, *H ros* rosette hemicryptophytes, *H scand* hemicryptophytes scandentia, *H scap* scapose hemicryptophytes, *He* helophytes, *NP* nanophanerophytes, *P caesp* caespitose phanerophytes, *P lian* lianous phanerophytes, *P scap* scapose phanerophytes, *P succ* succulent phanerophytes, *T rept* reptant therophytes, *T ros* rosette therophytes, *T scap* scapose therophytes, *Asiat.* Asiatic, *Atl.* Atlantic, *C-* Central, *Caucas.* Caucasic, *Circumbor.* Circumboreal, *Cosmopol.* Cosmopolite, *E* East, *Endem.* Endemic, *Eurimedit.* Euri-mediterranean, *Europ.* European, *Eurosib.* Eurosiberian, *Macaron.* Macaronesian, *Medit.* Mediterranean, *Mont.* Montane, *N* North, *Orient.* Oriental, *Orof.* Orofitic, *Paleotemp.* Paleotemperate, *S* South, *Saharo-Sind.* Saharo-Sindic, *Sic.* Sicilian, *Stenomedit.* Stenomediterranean, *Subtrop.* Subtropical, *Trop.* Tropical, *Turan.* Turanian, *W* West

^a^b-r—basal rosettes, bu.—bulbs, fl/infl—flowers/inflorescences, fl-b—flower buds, fr—portion of the fruits, le—leaves, ro—roots/tubers, st-j—stem juice and flower juice (nectar), t-s—tender shoots, including aerial parts, tender parts, tender stems, young shoots)

^b^Ra—raw, Co—cooked, Ra/Co—raw and cooked

^c^VVC—widely common, cited by more than 75% (*n* > 735) of the informants; VC—Very common, 50–75% (*n* = 490–735) of the informants; C—common, 20–50% (*n* = 196–490) of the informants; R—rare, 5–20% (*n* = 49–196) of the informants; VR—very rare, less than 5% (*n* < 49) of the informants

We compared our data with those gathered from the following sources: published Sicilian ethnobotanical surveys considering wild plants traditionally used in local cuisines [47, 48, 75–96]; the recent review concerning wild food plants used traditionally as vegetables in Italy [61] and other international papers [42–60]; ethnobotanical literature in which ethnobotanical studies focusing on wild food plants were conducted in Mediterranean areas and published in international journals, in particular, from Spain [63–72], Turkey [32–37], Morocco [73, 74], Croatia [28–30], Herzegovina [31], Cyprus [38], and Greece [39, 40], countries that have recognized the importance of the Mediterranean diet (see introduction). From these studies, we considered only the plants used as vegetables to make the data comparable with our reports. A multivariate analysis was performed to compare the affinity among the countries [111]. This analysis was carried out at the genus level because the comparisons among species are influenced by phytogeographical characteristics of each flora. A floristic binary matrix of 313 genera × 7 plots was classified through cluster analysis by using chord distance and UPGMA in the SYN-TAX Programme [112].

## Results and discussion

### Data on the plants recorded in Sicily

The data obtained after collecting information from the 980 people interviewed (Fig. 2) are reported in Table 1. There were 253 wild species belonging to 39 families and 128 genera used as vegetables that were recognized in our study, representing 7.78% of the Sicilian flora. The most represented were Asteraceae, with 39 genera and 94 taxa (37.15%); Brassicaceae, with 26 genera and 45 taxa (17.78%); Apiaceae, with 10 genera and 14 taxa (5.53%); Amaryllidaceae, with 2 genera and 8 taxa (3.16%); Malvaceae and Polygonaceae, with 7 taxa (2.76%) and 1 genus for each family; Plantaginaceae, with 1 genus and 6 taxa (2.37%); and Asparagaceae, Boraginaceae, and Caryophyllaceae, with 5 taxa and 1, 3, and 2 genera, respectively (Table 1).

**Fig. 4:**
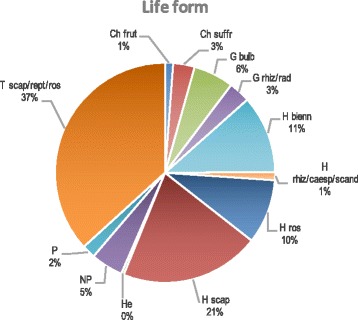
Biological spectrum (life-forms) of the taxa recorded

**Fig. 5:**
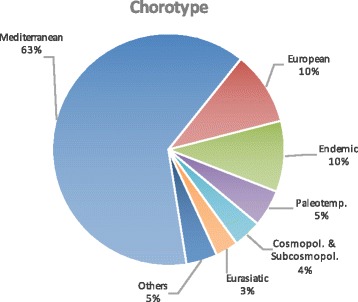
Biogeographic spectrum (chorotype) of the taxa

Considering life forms (Fig. 4), there were mainly hemicryptophytes (43.03%), therophytes (36.25%), and geophytes (9.16%). The main contingent of the taxa belongs to the Mediterranean chorotype (62.9%), 25 taxa (10%) are endemic and subendemic to Italian flora of which there are 10 endemic Sicilian taxa (Fig. 5). These wild vegetables commonly grow in uncultivated land, in the margins of cultivated fields or infesting them, and in pastures, garrigues, dry meadows, road edges, etc.; some can be gathered in the woods, ruins, cliffs, and slopes (Table 1).

**Fig. 6:**
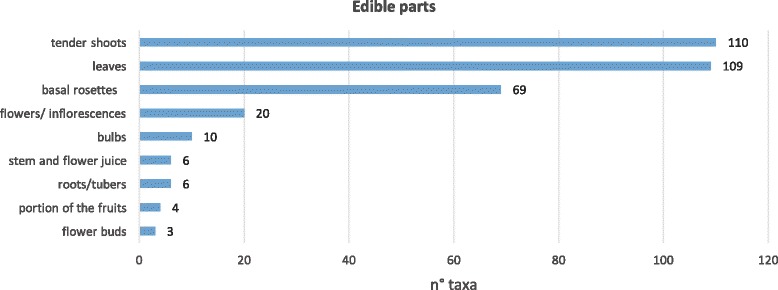
Parts of the wild vegetable used and their frequencies

The food uses of 26 plants were recorded for the first time in our present study (Table 2). The aerial parts of wild plants, including leaves (43.4%), tender shoots (43%), and basal rosettes (27.5%), are mainly utilized as vegetables, whereas the subterranean parts as a whole account for 6.4% (Fig. 6). For some vegetables, more parts are utilized (see Table 1).

**Table 2:** Summary of the results

	Taxa
Taxa recorded for the first time in Sicily.	*Bellis annua*, *B. perennis* var. *hybrida*, *B. perennis* var. *strobliana*, *B. sylvestris*, *Centaurea napifolia*, *Cichorium intybus* var. *glabratum*, *C. pumilum*, *Crepis sprengelii*, *C. vesicaria* subsp. *bivonana*, *C. vesicaria* subsp. *taraxacifolia*, *Leontodon cichoraceus*, *L. intermedius*, *L. muelleri*, *L. siculus*, *Tolpis umbellata*, *Xanthium strumarium* subsp. *strumarium*, *X. orientale* subsp. *italicum*, *Echium italicum* subsp. *siculum*, *Brassica rupestris* subsp. *hispida*, *Raphanus raphanistrum* subsp. *maritimus*, *Silene vulgaris* subsp. *commutata*, *Umbilicus horizontalis*, *U. rupestris*, *Gladiolus communis* subsp. *byzantinus*, *G. communis* subsp. *communis*, *Papaver rhoeas* var. *himerense.*
Taxa cited by 75% or more of the informant (VVC).	*Allium ampeloprasum*, *Foeniculum vulgare* subsp. *vulgare*, *Asparagus acutifolius, Cichorium intybus* var. *intybus*, *Reichardia picroides*, *Sonchus asper* subsp. *asper*, *S. oleraceus*, *S. tenerrimus*, *Borago officinalis*, *Brassica rapa* subsp. *campestris*, *Beta vulgaris* subsp. *vulgaris.*
Taxa rarely cited (VVR).	*Narcissus tazetta* subsp. *tazetta*, *Kundmannia sicula*, *Smyrnium olusatrum*, *S. perfoliatum*, *S. rotundifolium*, *Tordylium apulum*, *Taraxacum siculum*, *Brassica rupestris* subsp. *hispida*, *Rorippa sylvestris* subsp. *sylvestris*, *Papaver somniferum* subsp*. setigerum*, *Rumex crispus*, *Rubus ulmifolius*, *Sanguisorba minor* subsp. *minor*.
Wild vegetables found frequently in the markets.	*Foeniculum vulgare* subsp. *vulgare*, *Asparagus acutifolius*, *Cichorium intybus*, *Crepis* spp., *Cynara cardunculus* subsp. *cardunculus*, *Hypochaeris* spp., *Reichardia picroides*, *Sonchus* spp*.*, *Borago officinalis*, *Brassica rapa* subsp. *campestris*, *Eruca vesicaria*, *Hirschfeldia incana*, *Raphanus raphanistrum*, *Capparis spinosa* s.l., *Beta vulgaris* s.l., *Ruscus aculeatus*.
Wild vegetables found less frequently limited to small village markets.	*Allium ampeloprasum*, *A. nigrum*, *A. roseum*, *Asphodeline lutea*, *Centaurea calcitrapa*, *C. napifolia*, *Hyoseris radiata* and *H. scabra*, *Leontodon cichoraceus*, *Onopordum illyricum* s.l., *Scolymus grandiflorus*, *S. hispanicus* and *S. maculatus*, *Taraxacum* spp*.*, *Urospermum dalechampii* and *U. picroides*, *Brassica fruticulosa*, *B. nigra*, *Ruscus hypophyllum*.

Regarding the frequency of citation, only 13 taxa were cited by 75% or more of the interviewed people (VVC), 101 vegetable taxa were commonly gathered and consumed (VC and C), while 126 (49.8%) were rarely cited—ranging from 5 to 20% of informants (R category)—and 13 were very rarely cited (Tables 1 and 2). Among the taxa infrequently cited as vegetables, there are some Apiaceae believed to be toxic by our informants in some areas, some endemic species and other plants frequently used for other parts such as *Rubus ulmifolius* (for fruits). Another plant rarely cited is *Rumex crispus* that in some areas, it used as a vegetable, while in Villarosa-Enna, it is utilized for cigarette coatings [95]. Most of the reported vegetables are consumed cooked (238), with 159 only cooked and 79 both cooked and raw, whereas 94 are eaten raw and 15 are only eaten raw, generally used as snacks (*Chamaerops humilis*, *Carthamus lanatus* subsp. *lanatus*, *Rubus ulmifolius*), salads (*Eryngium campestre*, *Ridolfia segetum*, *Umbilicus horizontalis*, *U. rupestris*, *Rosa canina*, *R. sempervirens*), or for the juice of stems and flowers (*Gladiolus communis* s.l., *G. italicus*, *Lamium flexuosum*, *Veronica anagallis-aquatica*) (see Table 1).

Some vegetables should be eaten after cooking due to the presence of some thermolabile toxic substances [113] or bristly or stinging hairs or thorns, i.e., *Asphodelus ramosus* s.l., *Asphodeline lutea*, *Kundmannia sicula*, *Borago officinalis*, *Echium italicum* subsp. *italicum*, *E. italicum* subsp. *siculum*, *E. plantagineum*, *Opuntia ficus-indica* (the skins of the fruit), *Dioscorea communis, Leopoldia comosa*, * Iris tuberosa, Clematis vitalba, Smilax aspera*, *Lycium europaeum, Solanum americanum*, *Urtica* spp.

Most of the mentioned vegetables are collected only for family use and are not sold. Some species, on the other hand, are found rather frequently at the stands in the markets in both towns and rural villages, while some other vegetables are found less frequently and are limited to small villages. (Table 2). Wild vegetables are an important component of traditional food systems in Sicily as well as around the world [114]; in particular, they played a significant role in feeding the Sicilian population until the 1960s [75]. Later, with the massive movement of people from the country to towns, these vegetables have gradually been replaced with cultivated ones, whereas the non-cultivated vegetables have been increasingly less utilized in the daily diet. Their consumption represented and still represents the “hidden component” of the Mediterranean diet [24], the style of life that recommends the intake of a large amount of plant food in the diet (see introduction). As evident by the chorology, most of the gathered taxa belong to the Mediterranean element but more than 13% are taxa with wide geographic ranges (cosmopolite, subcosmopolite, paleotemperate, etc.). These latter plants usually grow in anthropogenic environments such as nitrophilous habitats, roadsides, ruins, etc. (Table 1).

The use of vegetables has a strong cultural value because it is linked to traditional Sicilian cooking, which includes preparation methods that enhance organoleptic qualities as well as healthiness. Wild vegetables still represent the main dishes at lunch or dinner (e.g., soups, omelets, salads) or special preparations during traditional festivities (i.e., wild thistles fried in batter for Christmas night or the traditional “*manciari di S. Giuseppe*” based on mixed vegetables). Moreover, the seasonality of non-cultivated vegetables permits variation of both the preparation of the main meals and the dishes accompanying the second courses. For example, in autumn, the bitter taste of *Brassica rapa* subsp. *campestris* (“*sinapi accupateddri*”) contrasts with the fat and sweet taste of grilled sausages, or *Beta vulgaris* s.l. leaves (*giri*) make the “*maccu di fave*” (fava bean puree) delicious. In the winter, a special dish is represented by *Allium ampeloprasum* fried bulbs (*purrietti*), while in the spring, an omelet with the tender shoots of *Asphodeline lutea* (*garufi*) is an appreciated main course. These typical dishes with wild vegetables are, therefore, elements of the cultural identity of Sicilian rural communities.

In our investigation, we identified 253 wild taxa utilized as vegetables. This is a very high number, justified by the fact that Sicily has been a crossroad of cultures because of its geographical position, and several historical colonizations by Mediterranean and European peoples, such as the Phoenicians, Greeks, Romans, Turks, Arabs, French, and Spanish, occurred on the island. Every ancient culture brought its own food traditions, which have been passed down through the years. Luckily, although the use of wild vegetables in the diet has been considerably reduced, the long-established cuisine using these vegetables is still quite alive in many rural villages in Sicily, as it occurs in southern Italy [24, 43, 44] and in other Mediterranean countries [31, 32, 73, 74]. In Sicily, the rural areas are still inhabited by a significant number of farmers. Recently, agricultural activities using techniques that are more respectful of both the environment and traditional biodiversity (the use of ancient cultivars of cereal, fruit trees, etc.) have been increasing. This trend allows the maintenance of ancient and well-established food traditions that also consider also wild plants.

### Comparing Sicilian data with other areas

**Fig. 7:**
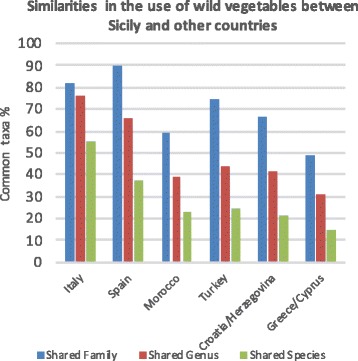
Comparison of vegetable taxa among Sicily and other Mediterranean areas

Comparing our Sicilian findings with previous studies and studies in other countries within the Mediterranean area (Table 3), we detected 253 vegetable taxa. For Sicily, previous studies by Lentini and Venza [47] and Pasta et al. [48] reported 188 taxa (48 families, 126 genera) and 254 taxa (38 families, 148 genera), respectively. They also included taxa used for edible fruits, seeds, and aromatic uses or seasonings; for this reason, we share 132 taxa with Lentini and Venza [47] and 179 with Pasta et al. [48]. Recently, in their extensive review, Guarrera and Savo [61] have described 276 taxa (40 family and 161 genera) in Italy, including 11 seasoning plants (such as *Thymus*, *Mentha*, *Origanum*, and *Laurus,* which are excluded from Table 3). The number of taxa detected in Sicily is similar to the overall data reported from several areas in Spain, but it is higher than the number obtained from Turkey and Morocco, as well as from smaller countries in the eastern Mediterranean region. Several families and genera of collected vegetables are shared between Sicily and Italy (82% of families and 77% of genera) and between Sicily and Spain (90% of families and 66% of genera), while less than 50% are in common with other countries (Fig. 7). As expected, the number of shared species decreases significantly, since each region presents its own floristic particularities; in this study, for example, we recorded 25 endemic and subendemic plants (Table 1). Only Agavaceae and Cactaceae are reported in Sicily as naturalized taxa. The use of *Agave americana* was already cited by Lentini and Venza [47], and *Opuntia ficus-indica* was cited [47, 48] for its edible fruit, while we report this taxon for the use of the peel (epicarp and mesocarp) of the fruit as a vegetable (see below). Edible species among the Iridaceae and the Juncaceae, apart from in Sicily, were recorded only in Spain and Morocco, respectively.

**Table 3:** Comparison among Sicilian data and other Mediterranean countries (only the vegetable use was considered)

	Sicily	Italy^a^	Spain^b^	Turkey^c^	Morocco^d^	Croatia/Herzegovina^e^	Cyprus/Greece^f^
No. of families	39	40	53	36	37	32	23
No. of genera	128	162	158	97	98	74	57
No. of taxa	253	299	277	151	158	98	76

Data from (a) [42–62], (b) [63–72], (c) [32–37], (d) [73, 74], (e) [28–31], (f) [38–40]

Considering the total taxa recorded in the other countries (Table 3), only Spain and Italy utilize more plants than Sicily as vegetables—277 and 299, respectively, which represent 3.96 and the 3.89% of their entire floras [106, 115]. In Morocco, the reported taxa reach 4.1% of the flora [73], while in Turkey, only 1.3% was reported [116], which is probably an underestimation, considering the high plant diversity of the Turkish regions. The data obtained from the comparison highlight some differences in the use of taxa both at family and genus levels (Table 4). Some families recorded in the compared Mediterranean countries are not employed in Sicily as vegetables, and there are some edible genera fairly recurrent in other countries that are not recorded in Sicily (Table 4). In some cases, this occurs because some taxa do not belong to the Sicilian flora, i.e., *Neurada procumbens* L. (Neuradaceae), *Sesamum alatum* Thonn. (Pedaliaceae), *Balanites aegyptiaca* (L.), Delile (Zygophyllaceae), *Glossonema boveanum* (Decne.) Decne. and *Leptadenia pyrotechnica* (Forssk.) Decne. (Apocynaceae), *Gymnosporia senegalensis* (Lam.) Loes. (Celastraceae), and *Cistanche phelypaea* (L.) Cout. (Orobanchaceae), gathered in Morocco for various uses [73, 74]. *Cistus ladanifer* L. (Cistaceae) and *Vaccinium myrtillus* L. (Ericaceae) are used in Spain for flower juice [68] and the young shoots [63], respectively. *Zygophyllum fabago* L. (Zygophyllaceae) is used for the flowers in Sardinia [62] and *Linum hirsutum* L. s.l. is used for flower juice in Afyonkarahisar in Turkey [37]. In other cases, although the taxa are also distributed in Sicily, they are not traditionally consumed as vegetables. For example, peeled bulbs of *Colchicum montanum* L. (Colchicaceae) and young shoots of *Vitis vinifera* subsp. *sylvestris* (C.C. Gmel.) Hegi (Vitaceae) are consumed in Spain as well as species belonging to the genera *Aegilops* and *Stipa* of Poaceae that are used as vegetables [68–70]. Among the Crassulaceae, the leaves of *Sedum album* L., *S. sediforme* (Jacq.) Pau are eaten raw as a snack or in salads or stewed in Spain [68]. Also in Turkey, the use of *Sedum* (*S. rubens* L.) as a vegetable is reported [32, 34]. *Bryonia cretica* subsp. *dioica* (Jacq.) Tutin (Cucurbitaceae) is traditionally used in Spain [66, 68, 69] and in Herzegovina [31]. In Turkey, cooked or raw (roasted or in a salad) leaves of *Fumaria officinalis* L. (Fumariaceae) [32, 35, 36] are eaten as well as cooked (stuffed, meal, roasted) leaves of *Arum maculatum* L. (Araceae) [32, 35]. Additionally, in Croatia and Herzegovina, *Arum italicum* Mill. cooked leaves were utilized as famine food during the war era [30, 31], and the traditional use of *Knautia integrifolia* (Honck. ex L.) Bertol. (Caprifoliaceae) is reported for Krk island in Croatia [30]. Young shoots *of Lythrum salicaria* L. (Lythraceae) are consumed only in the Calabria region (Italy), in which the use of young basal leaves of *Reseda alba* L. (Resedaceae) is also reported. *Oenothera biennis* L. (boiled root), *Epilobium angustifolium* L., and *Epilobium montanum* L. (young shoots) belonging to the Onagraceae are eaten in the northern Italian region [61]. Although taxa belonging to *Erodium*, *Anchusa*, *Scandix*, and *Campanula* (growing also in Sicily) are commonly eaten in almost all Mediterranean countries, they were not recognized as wild vegetables by our informants.

**Table 4:** Comparison among Sicilian data and other Mediterranean countries

	Taxa
Families recorded in the compared Mediterranean countries not employed in Sicily for their vegetable taxa	Aizoaceae, Anacardiaceae, Araceae, Apocynaceae, Aristolochiaceae, Campanulaceae, Cannabaceae, Celastraceae, Cistaceae, Colchicaceae, Convolvulaceae, Cucurbitaceae, Cymodoceaceae, Cynomoriaceae, Cytinaceae, Equisetaceae, Ericaceae, Euphorbiaceae, Fumariaceae, Geraniaceae, Hypolepidaceae, Liliaceae, Linaceae, Lythraceae, Neuradaceae, Onagraceae, Orobanchaceae, Pedaliaceae, Plumbaginaceae, Poaceae, Resedaceae, Rubiaceae, Saxifragaceae, Typhaceae, Ulmaceae, Violaceae, Vitaceae, Zygophyllaceae.
Edible genera fairly recurrent in other countries not recorded in Sicily	*Erodium* (in all except Cyprus), *Anchusa* (in all except Italy), *Scandix* (in all except Morocco), *Campanula* (in all except Croatia/Herzegovina), *Convolvulus* (in Spain, Morocco, Croatia, Cyprus), *Limonium* (in Spain, Turkey, Morocco, Cyprus), *Atriplex* (in Italy, Spain, Morocco), *Cirsium* (in Italy, Spain, Turkey).
Taxa collected and eaten in Sicily and in all investigated countries	*Foeniculum vulgare* subsp. *vulgare*, *Asparagus acutifolius*, *Cichorium intybus*, *Glebionis coronaria*, *Sonchus oleraceus*, *Borago officinalis*, *Capsella bursa-pastoris*, *Silene vulgaris* subsp. *vulgaris*, *Beta vulgaris*, *Malva sylvestris* subsp. *sylvestris*, *Papaver rhoeas* var. *rhoeas*, *Portulaca oleracea* subsp. *oleracea*
Taxa commonly collected in Sicily and in five other compared countries	*Allium ampeloprasum*, *Crithmum maritimum*, *Smyrnium olusatrum*, *Cynara cardunculus* subsp. *cardunculus*, *Scolymus hispanicus*, *Sonchus asper* subsp. *asper*, *Eruca vesicaria* subsp*. sativa, Nasturtium officinale*, *Sinapis alba* subsp. *alba*, *Chenopodium album*, *Rumex pulcher* subsp. *pulcher*, *Urtica dioica.*
Taxa commonly collected in Sicily and in four other compared countries	*Amaranthus retroflexus*, *Apium nodiflorum*, *Eryngium campestre*, *Bellis perennis* var*. perennis*, *Chondrilla juncea*, *Lactuca serriola, Scolymus maculatus*, *Silybum marianum*, *Tragopogon porrifolius* subsp. *porrifolius*, *Urospermum picroides*, *Brassica nigra*, *Rapistrum rugosum*, *Sinapis arvensis*, *Sisymbrium officinale*, *Capparis spinosa* subsp*. spinosa*, *Stellaria media*, *Beta vulgaris* subsp*. maritima*, *Dioscorea communis*, *Oxalis pes-caprae*, *Plantago lanceolata*, *Plantago major* subsp. *major*, *Rumex crispus, Smilax aspera.*

Moreover, in our study, we observed that some species thought to be inedible in Sicily are eaten as vegetables in other countries; for example, *Mercurialis annua* L. is used in a soup in Turkey [32, 35] as well as *Euphorbia chamaesyce* L. [36] and *Euphorbia helioscopia* L. [35]. Several species of *Euphorbia* are also consumed in Morocco (*Euphorbia granulata* Forssk., *Euphorbia balsamifera* Aiton*, Euphorbia officinarum* susbp*. echinus* (Hook.f. & Coss.) Vindt*, Euphorbia regis jubae* J.Gay*, Euphorbia resinifera* O.Berg.). Guarrera and Savo [61] report the use of *Chrozophora tinctoria* (L.) A. Juss. and *Equisetum arvense* L. in Italy. In Spain, the edible use of *Pteridium aquilinum*, assumed to be very harmful to human health in Sicily, is reported. [63, 70]. The use of *Ferula communis* L. was detected in Morocco [73, 74]. In Sicily, we found a report of the sporadic consumption of inflorescences for the territory of Bronte [48, 89]. The plant is notoriously toxic and dangerous to animals, especially if eaten fresh [117, 118]. Its sporadic use was also confirmed by Biscotti and Pieroni [24] for Apulia (Italy). In our research, none of the interviewed people mentioned a current or previous food use of this plant.

**Fig. 8:**
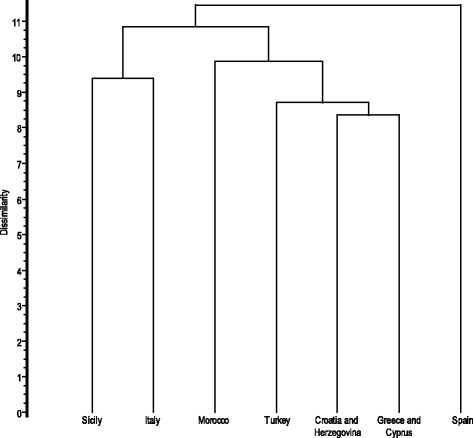
UPGMA cluster analysis showing the dissimilarity at genus level

Cluster analysis based on the current state of ethnobotanical knowledge of vegetable uses at the genus level shows a clustering reflecting the phytogeographical affinities of floras. The dendrogram depicts four main groups: (1) Spain, the country more investigated for ethnobotanical aspects, differs due to the Mediterranean-Atlantic chorological characteristics of its flora; (2) eastern Mediterranean countries; (3) Morocco, characterized by a sub-Saharan component of the flora; and (4) Sicily and Italy, as expected, because Sicily shares the highest number of genera with Italy (Fig. 8). Multivariate analysis revealed that the cultural diversities, in term of traditional uses of plants, are expressions of the biological diversities of the countries.

The families with the highest number of vegetables are Asteraceae, Brassicaceae, and Apiaceae. A great number of taxa of Amaryllidaceae, Malvaceae, Polygonaceae, Plantaginaceae, Asparagaceae, Boraginaceae, and Caryophyllaceae are also collected as vegetables in almost all regions [28–74]. In Sicily, we listed the highest number of Asteraceae and Brassicaceae taxa (species and subspecies), but at the genus level in Spain and Italy, the number is greater for Asteraceae. In Sicily, the contingent of Brassicaceae collected as vegetables was the highest in comparison with all other compared countries, including Italy, while the number of the taxa belonging to the Apiaceae was slightly smaller. For Boraginaceae, we reported five species belonging to three genera (see Table 1), but more taxa were recorded in Spain (*Anchusa azurea* Mill., *A. undulata* L., *Borago officinalis*, *Buglossoides arvensis* (L.) I.M. Johnst., *Echium creticum* L., *E. plantagineum*, *E. vulgare* L., *Lithodora fruticosa* (L.) Griseb), Morocco (*Anchusa azurea, Borago officinalis, Echium plantagineum, Heliotropium crispum* Desf.*, Trichodesma africanum* (L.) Sm.*, T. calcaratum* Coss. & Batt.), and Turkey [*Anchusa azurea, A. leptophylla* Roem. & Schult. subsp. *leptophylla, A. undulata* subsp. *hybrida* (Ten.) Bég.*, Borago officinalis, Cerinthe major* L. subsp. *major, Echium italicum, Paracaryum aucheri* (DC. & A.DC.) Boiss.*, Trachystemon orientalis* (L.) D.Don]. Amaryllidaceae, Asparagaceae, and Polygonaceae comprise several species traditionally collected and eaten by people, but they only belong to one or two genera in Sicily (Table 1) as well as in the compared Mediterranean areas. For Capparaceae, the case of Morocco is remarkable, where there are five edible taxa belonging to four different genera (*Cadaba farinosa* Forssk., *Capparis spinosa* L. subsp. *spinosa, C. decidua* (Forssk.) Edgew., *Cleome amblyocarpa* Barratte & Murb., *Maerua crassifolia* Forssk.).

Among the species reported in Table 1, 72 are eaten only in Sicily (marked with an asterisk*), while 12 are collected and eaten in Sicily and in all the investigated countries (Table 4). Twelve are very commonly collected in Sicily and in five other compared countries, while 23 are commonly collected in Sicily and in four other compared countries (Table 4).

Comparing the data collected for Sicily with those of a study on gathered Mediterranean food plants [119] in which 16 species (*Allium ampeloprasum*, *Arbutus unedo* L., *Asparagus acutifolius*, *Borago officinalis*, *Cichorum intybus*, *Chondrilla juncea*, *Crataegus monogyna* Jacq., *Foeniculum vulgare*, *Malva sylvestris*, *Nasturtium officinale*, *Rubus ulmifolius*, *Papaver rhoeas*, *Portulaca oleracea*, *Scolymus hispanicus*, *Silene vulgaris*, and *Sonchus oleraceus*) were considered of widespread use (˃ 33% of 62 zones), we noted that 14 are also utilized in Sicily as vegetables, with the exception of *Arbutus unedo* and *Crataegus monogyna* whose fruits, however, are harvested and consumed. In Herzegovina, wild plants are still an important source of nutrition for many people during the spring, and the resilience of the knowledge and use of wild vegetables is rather high (69–86%) [31]. Among the most commonly used vegetables, some taxa are also frequently collected in Sicily (*Dioscorea communis*, *Sonchus* spp., *Allium* spp., *Papaver roehas*), while different taxa of the genus *Silene* are eaten with respect to those consumed in Sicily. In various regions of Croatia, as in Sicily, *Asparagus acutifolius*, *Crepis* spp., *Cichorium intybus*, *Dioscorea communis*, *Sonchus* spp., *Allium ampeloprasum*, *Picris echioides*, *Foeniculum vulgare*, *Taraxacum officinale*, *Urospermum picroides*, *Beta vulgaris*, are the best-known vegetables, and together with *Bunias erucago*, *Papaver roehas*, and *Urtica* spp., they are commonly sold in the markets; some are sold mixed, others in separate bunches (*Asparagus, Dioscorea, Foeniculum*) [28–30]. Although in Spain the greatest number of species used as vegetables belongs to Asteraceae, *Nasturtium officinale* (sub *Rorippa nasturtium-aquaticum* (Moench) Beck) is the species whose consumption was cited most often [67]. Also very popular are *Asparagus acutifolius*, *Scolymus hispanicus*, *Silene vulgaris*, *Cichorium intybus*, *Foeniculum vulgare*, *Portulaca oleracea*, and *Montia fontana* L., *Urtica dioica* in the Madrid Province [66]. Peeled young shoots of *Rubus ulmifolius* are eaten as snacks as well as in Sicily, and in the Basque area, *Pteridium aquilinum* (L.) Kuhn is also consumed [63]. In Turkey, the rich biological and cultural diversities affect the traditional use of plants and are reflected in the rich Turkish cuisine [32]. In the Aegean region of Turkey, *Rumex* and *Erodium* (not cited by our informants for Sicily) are the most represented genera, while the best represented families are *Asteraceae* and *Boraginaceae* (19 taxa), and the use of several taxa of *Malva* has been reported as well in Sicily [32]. The most frequently consumed “greens” and the favorite food in the Bodrum area [34] are very similar to what we detected in Sicily: *Allium ampeloprasum*, *Foeniculum vulgare*, some Brassicaceae (*Sinapis*, *Brassica*, *Raphanus*), *Asparagus acutifolius*, *Dioscorea communis*, *Smilax aspera*, *Scolymus hispanicus*, and *Onopordon illyricum*. In Morocco, the consumption of wild plants is linked with the seasonality, the regional variability, and urban-rural differences. Several vegetables are commonly sold in local markets and on roadsides, such as *Asparagus* spp., *Malva* spp., *Portulaca oleracea*, and *Scolymus hispanicus* [73, 74]. These taxa are frequently eaten in Sicily but rarely found in local markets, except for *Asparagus* turions (see Table 2). The greatest affinity between Sicilian reports and those from Italy is shown in the dendrogram (Fig. 8), even if only 139 out of the 253 Sicilian vegetables are cited on the Italian list [61]. *Smilax aspera*, *Cyperus esculentus*, and several species of *Malva* and *Leontodon* were not reported for Italy. Among the most cited Italian taxa, *Cichorium intybus*, *Sonchus* spp., and *Reichardia picroides* were also very commonly cited by people in Sicily. *Taraxacum campylodes* G.E. Haglund was the most cited in Italy but not in Sicily. More similarity resulted with vegetable uses between Sicily and southern Italy [24].

In Sicily and other Mediterranean countries, the maintenance of the traditional market system, where people can find wild vegetable, is useful to preserve the habitual consumption of traditional food [74]. Moreover, the livelihood of rural people may depend not only on agricultural activity but also on the utilization of natural resources as wild vegetables that play a significant role in the human diet [33].

### Peculiarities of the use of some species in Sicily

**Fig. 9:**
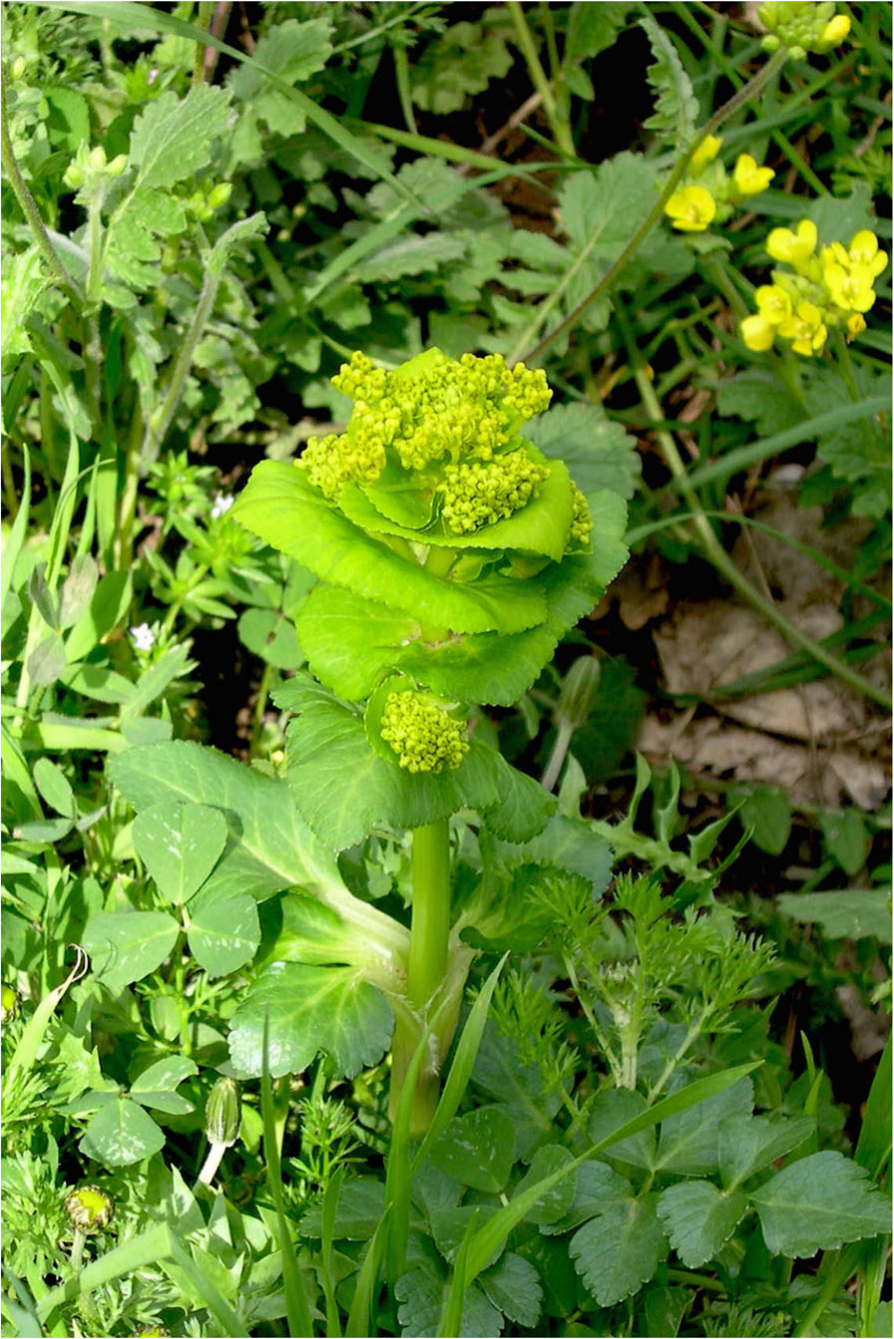
*Smyrnium rotundifolium* consumed in the Isnello village (Madonie Mountains)

**Fig. 10:**
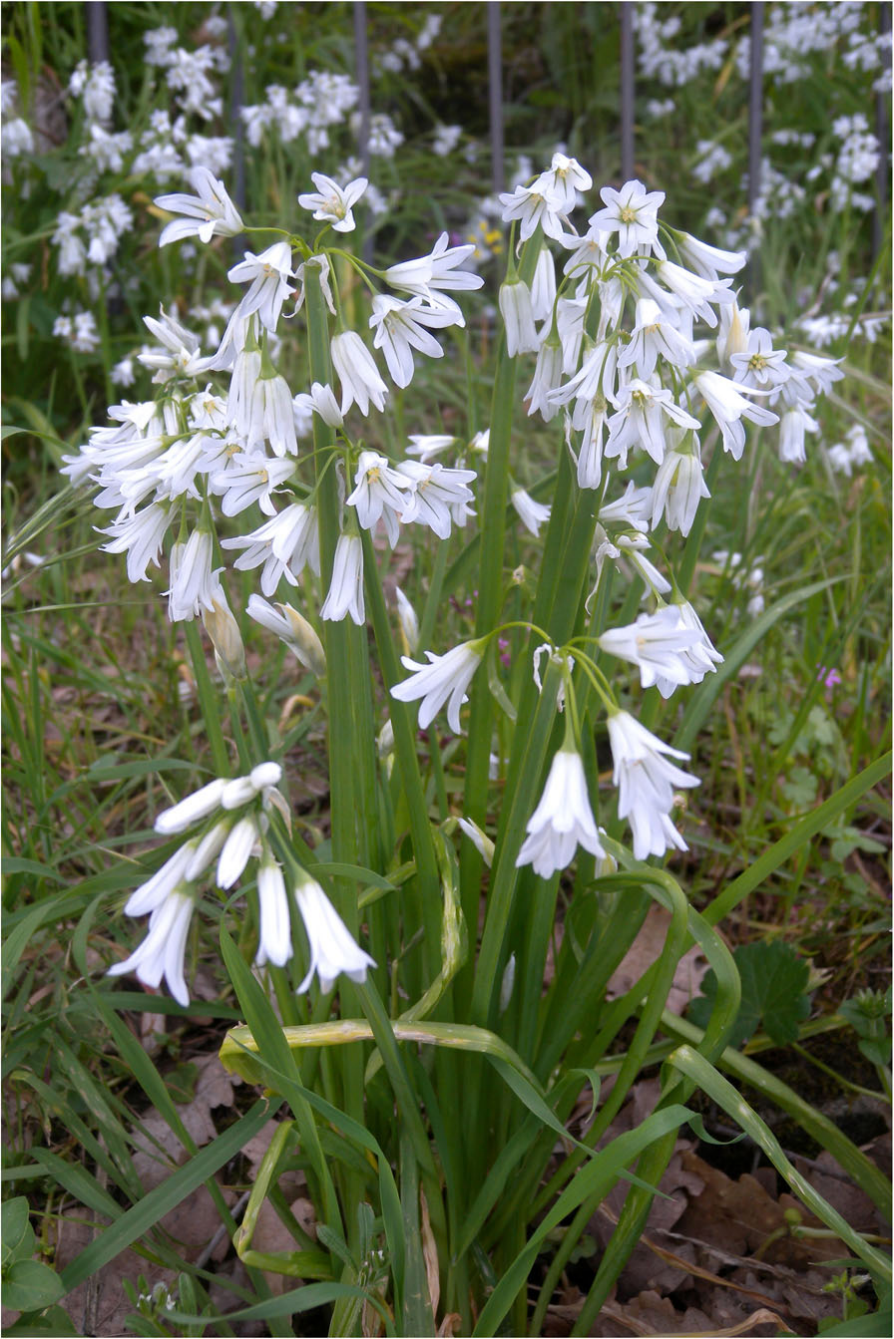
*Allium triquetrum* employed in traditional dishes

Among the surveyed species, some have a particular use and are limited to small local contexts, i.e., *Smyrnium rotundifolium* (Fig. 9), *Opuntia ficus-indica* (peel of the fruit), *Kundmannia sicula*, *Carlina gummifera*, *Centaurea calcitrapa*, *Onopordum* species, and *Allium triquetrum* (Fig. 10). In particular, in Sicily, *Smyrnium rotundifolium* is gathered and consumed only in the village of Isnello (approximately 2000 inhabitants, in the Madonie mountains near Palermo), where it is stored after being boiled in water and vinegar and eaten as an appetizer or used for flavoring salads. The use of this taxon was only also reported in Sardinia [120]. An uncommon use limited to some small rural communities of the Madonie Mountains (Palermo) is that of the peels of the prickly pear fruit (*Opuntia ficus-indica*), which are sun-dried and used during the winter, after being boiled, floured, and fried in extra-virgin olive oil. The consumption of *Kundmannia sicula* is restricted to a few villages of the Nebrodi and Madonie areas, where it is boiled together with other non-cultivated vegetables or employed for flavoring “*macco di fave*,” a puree of dried fava beans that is cooked slowly and to which *Kundmannia* (instead of the common fennel) is added at the end of cooking to enhance the taste. *Carlina gummifera* (locally called *“masticogna”*, see Table 1) is currently used in a few rural communities, where the fleshy receptacles of the capitula are consumed raw (rarely) or boiled and stewed. Its use in the territory of Tusa (ME) is noteworthy, where it is traditionally prepared in a sauce based on sterile sheep meat and the heads of this plant (*sucu di pecura strippa e masticogna*). *Centaurea calcitrapa* is a popular vegetable, especially in the mountain villages of the Madonie region, where the basal rosette is utilized between spring and autumn, when it is boiled and seasoned with extra-virgin olive oil or used to season pasta together with fresh ricotta (*pasta ccu l’apròcchi ri picurara e ricotta frisca*). *Onopordum* sp. is a vegetable traditionally used in various localities of Sicily, but recently, it has become a staple of the cuisine of restaurants in the town of Castelbuono (Palermo). The petioles and foliar rachis, after removing the thorns, are boiled and then cooked in a pan with garlic, breadcrumbs, tomato sauce, oil, and chili or used to prepare a particular seasoning for pasta (*sucu di “napurdi”*) by slowly cooking pieces of *Onopordum*, already boiled, in tomato sauce and extract. *Allium triquetrum* is used in place of *Allium sativum*. Both the cloves and the leaves are employed to prepare “*spaghetti with agliotta*,” which is seasoned with extra-virgin olive oil, pepper, and pecorino cheese. Lastly, much curiosity has been aroused by the consumption, albeit limited, of the leaves of *Umbilicus rupestris* and *U. horizontalis*—known for use in traditional medicine [121]—in salads with other typical seasonal vegetables.

## Conclusion

Wild vegetables in Sicily still represent an important resource, as they can enrich the table with strong (bitter) or delicate flavors that give a unique taste and experience: rustic, primitive, rough but genuine, and able to reconcile “man with nature.” In addition to the vegetables well-known by the population (borage, wild beets, chicory, thistles, etc.), some vegetables are almost unknown to most people, i.e., the so-called ancient vegetables, including *Onopordum* spp., *Centaurea calcitrapa*, *Nasturtium officinale*, *Scolymus* spp., and *Smyrnium rotundifolium*.

Wild vegetables, with the traditions, customs, and practices surrounding them, are a part of the Sicilian cultural heritage, which unfortunately every day is at risk of disappearing under the pressure of globalization. This situation may, in a few decades, lead to the loss of the knowledge acquired throughout the centuries by generations of farmers, herders, foresters and other people who lived closely together with nature (our main informants, see Fig. 3). Such a loss would be very heavy because it would deprive the population of a food source of considerable interest from a qualitative point of view. Non-cultivated vegetables are rich in nutritional components that are often present in smaller quantities in species of cultivated varieties, which are selected for their high manufacturing yields. In times of possible food shortages, the population would no longer be able to identify the food resources available.

In recent years, there has been a renewed interest in non-cultivated vegetables, for both cognitive and consumption reasons, because of the growing demand for healthy foods related to a specific territory that is connected to identity. Wild vegetables are, in fact, the best ambassadors of the site in which they live. They are able to please tourists through the many local culinary preparations, expressing a solid and layered cultural tradition. The latter represents the real added value of a raw material that is obtained in an environment unique in its biological characteristics, soil, climate, and history, and which can be considered as the most expressive and symbolic cradle of the Mediterranean diet.

## Abbreviations

Asiat.: Asiatic
Atl.: Atlantic
b-r: Basal rosettes
bu: Bulbs
C-: Central
C: Common; 20–50% (*n* = 196–490) of the informants
Co: Cooked
Caucas.: Caucasic
Ch frut: Fruticose chamaephytes
Ch suffr: Suffruticose chamaephytes
Circumbor.: Circumboreal
Cosmopol.: Cosmopolite
E: East
Endem.: Endemic
Eurimedit.: Euri-mediterranean
Europ.: European
Eurosib.: Eurosiberian
fl/infl: Flowers/inflorescences
fl-b: Flower buds
fr: Portion of the fruits
G bulb: Bulbous geophytes
G rad: Root-budding geophyte
G rhiz: Rhizome-geophytes
H bienn: Biennial hemicryptophytes
H caesp: Caespitose hemicryptophytes
H rhiz: Rhizomatous hemicryptophytes
H ros: Rosette hemicryptophytes
H scand: Hemicryptophytes scandentia
H scap: Scapose hemicryptophytes
He: Helophytes
le: Leaves
Macaron.: Macaronesian
Medit.: Mediterranean
Mont.: Montane
N: North
NP: Nanophanerophytes
Orient.: Oriental
Orof.: Orofitic
P caesp: Caespitose phanerophytes
P lian: Lianous phanerophytes
P scap: Scapose phanerophytes
P succ: Succulent phanerophytes
Paleotemp.: Paleotemperate
R: Rare; 5–20% (*n* = 49–196) of the informants
Ra: Raw
Ra/Co: Raw and cooked
ro: Roots/tubers
Saharo-Sind.: Saharo-Sindic
Sic.: Sicilian
S: South
Stenomedit.: Stenomediterranean
st-j: Stem juice and flower juice (nectar)
Subtrop.: Subtropical
T rept: Reptant therophytes
T ros: Rosette therophytes
T scap: Scapose therophytes
Trop.: Tropical
t-s: Tender shoots, including aerial parts, tender parts, tender stems, young shoots
Turan.: Turanian
VC: Very Common; 50–75% (*n* = 490–735) of the informants
VR: Very rare; less than 5% (*n* < 49) of the informants
VVC: Widely common; cited by more than 75% (*n* > 735) of the informants
W: West

## References

[1] Willett WC, Sacks F, Trichopoulou A, Drescher G, Ferro-Luzzi A, Helsing E (1995). Mediterranean diet pyramid: a cultural model for healthy eating. Am J Clin Nutr.

[2] Sofi F, Cesari F, Abbate R, Gensini GF, Casini A (2008). Adherence to Mediterranean diet and health status: meta-analysis. BMJ.

[3] Sofi F, Macchi C, Abbate R, Gensini GF, Casini A (2013). Mediterranean diet and health. Biofactors.

[4] UNESCO: Mediterranean diet. 2013. http://www.unesco.org/culture/ich/en/RL/mediterranean-diet-00884. Accessed 20 Apr 2017.

[5] Keys A (1980). Seven countries: a multivariate analysis of death and coronary heart disease.

[6] Martinez-Gonzalez MA, Bes-Rastrollo M, Serra-Majem L, Lairon D, Estruch R, Trichopoulou A (2009). Mediterranean food pattern and the primary prevention of chronic disease: recent developments. Nutr Rev.

[7] Sofi F, Abbate R, Gensini GF, Casini A (2010). Accruing evidence on benefits of adherence to the Mediterranean diet on health: an updated systematic review and meta-analysis. Am J Clin Nutr.

[8] Buckland G, Gonzalez CA, Agudo A, Vilardell M, Berenguer A (2009). Adherence to the Mediterranean diet and risk of coronary heart disease in the Spanish EPIC cohort study. Am J Epidemiol.

[9] Hu EA, Toledo E, Diez-Espino J, Estruch R, Corella D (2013). Lifestyles and risk factors associated with adherence to the Mediterranean diet: a baseline assessment of the PREDIMED trial. PLoS One.

[10] Giacosa A, Barale R, Bavaresco L, Gatenby P, Gerbi V, Janssens J (2013). Cancer prevention in Europe: the Mediterranean diet as a protective choice. Eur J Cancer Prev.

[11] Trichopoulou A, Costacou T, Bamia C, Trichopoulos D (2003). Adherence to a Mediterranean diet and survival in a Greek population. N Engl J Med.

[12] Estruch R, Ros E, Salas-Salvadó J, Covas MI, Corella D, Arós F, Gómez-Gracia E, Ruiz-Gutiérrez V, Fiol M, Lapetra J (2013). Primary prevention of cardiovascular disease with a Mediterranean diet. N Engl J Med.

[13] Keys A, Mienotti A, Karvonen MJ, Aravanis C, Blackburn H, Buzina R (1986). The diet and 15-year death rate in the seven countries study. Am J Epidemiol.

[14] Naska A, Trichopoulou A (2014). Back to the future: the Mediterranean diet paradigm. Nutr Metab Cardiovasc Dis.

[15] Klein BP, Kurilich AC (2000). Processing effects on dietary antioxidants from plant foods. HortSci.

[16] Poiroux-Gonord F, Bidel LPR, Fanciullino AL, Gautier H, Lauri-Lopez F, Urban L (2010). Health benefits of vitamins and secondary metabolites of fruits and vegetables and prospects to increase their concentrations by agronomic approaches. J Agric Food Chem.

[17] Nahak G, Suar M, Sahu RK (2014). Antioxidant potential and nutritional values of vegetables: a review. Res J Med Plant.

[18] Nomikos T, Detopoulou P, Fragopoulou E, Pliakis E, Antonopoulou S (2007). Boiled wild artichoke reduces postprandial glycemic and insulinemic responses in normal subjects but has no effect on metabolic syndrome patients. Nutr Res.

[19] Renna M, Rinaldi VA, Gonnella M (2015). The Mediterranean diet between traditional foods and human health: the culinary example of Puglia (southern Italy). Int J Gastronomy Food Sci.

[20] Visioli F, Galli C (2001). The role of antioxidants in the Mediterranean diet. Lipids.

[21] Pitsavos C, Panagiotakos D, Tzima N, Chrysohoou C, Economou M, Zampelas A, Stefanadis C (2005). Adherence to the Mediterranean diet is associated with total antioxidant capacity in healthy adults: the ATTICA study. Am J Clin Nutr.

[22] Estruch R, Martínez-González MA, Corella D, Basora-Gallisá J, Ruiz-Gutiérrez V, Covas MI, Fiol M, Gómez-Gracia E, López-Sabater MC, Escoda R (2009). Effects of dietary fibre intake on risk factors for cardiovascular disease in subjects at high risk. J Epidemiol Community Health.

[23] Luczaj L, Pieroni A, Tardio J, Pardo-de-Santayana M, Soukand R, Svanberg I, Kalle R (2012). Wild food plant use in 21st century Europe: the disappearance of old traditions and the search for new cuisines involving wild edibles. Acta Soc Bot Pol.

[24] Biscotti N, Pieroni A (2015). The hidden Mediterranean diet: wild vegetables traditionally gathered and consumed in the Gargano area, Apulia, SE Italy. Acta Soc Bot Pol.

[25] Heinrich M, Leonti M, Nebel S, Peschel W (2005). “Local food—nutraceuticals”. An example of a multidisciplinary research project on local knowledge. J Physiol Pharmacol.

[26] Conforti F, Marrelli M, Carmela C, Menichini F, Valentina P, Uzunov D (2011). Bioactive phytonutrients (omega fatty acids, tocopherols, polyphenols), in vitro inhibition of nitric oxide production and free radical scavenging activity of non-cultivated Mediterranean vegetables. Food Chem.

[27] Marrelli M, Loizzo MR, Nicoletti M, Menichini F, Conforti F (2014). In vitro investigation of the potential health benefits of wild Mediterranean dietary plants as anti-obesity agents with α-amylase and pancreatic lipase inhibitory activities. J Sci Food Agric.

[28] Łuczaj Ł, Zovkokoncic M, Milicevic T, Dolina K, Pandza M (2013). Wild vegetable mixes sold in the markets of Dalmatia (southern Croatia). J Ethnobiol Ethnomed.

[29] Dolina K, Łuczaj Ł (2014). Wild food plants used on the Dubrovnik coast (south-eastern Croatia). Acta Soc Bot Pol.

[30] Dolina K, Jug-Dujaković M, Łuczaj Ł, Vitasović-Kosić I (2016). A century of changes in wild food plant use in coastal Croatia: the example of Krk and Poljica. Acta Soc Bot Pol.

[31] Łuczaj Ł, Dolina K (2015). A hundred years of change in wild vegetable use in southern Herzegovina. J Ethnopharmacol.

[32] Dogan Y (2012). Traditionally used wild edible greens in the Aegean region of Turkey. Acta Soc Bot Pol.

[33] Dogan Y, Ugulu I, Durkan N (2013). Wild edible plants sold in the local markets of Izmir. Turkey Pak J Bot.

[34] Ertuğ F (2004). Wild edible plants of the Bodrum area (Muğla, Turkey). Turk J Bot.

[35] Dogan Y, Baslar S, Ay G, Mert HH (2004). The use of wild edible plants in western and central Anatolia (Turkey). Econ Bot.

[36] Ozbucak TB, Kutbay H, Akcın OE (2006). The contribution of wild edible plants to human in the Black Sea region of Turkey. Ethnobot Leaflets.

[37] Arı S, Temel M, Kargıoğlu M, Konuk M (2015). Ethnobotanical survey of plants used in Afyonkarahisar-Turkey. J Ethnobiol Ethnomed.

[38] Della A, Paraskeva-Hadjichambi D, Hadjichambis A (2006). An ethnobotanical survey of wild edible plants of Paphos and Larnaca countryside of Cyprus. J Ethnobiol Ethnomed.

[39] Brussell D (2004). Medicinal plants of Mt. Pelion, Greece. Econ Bot.

[40] Psaroudaki A, Dimitropoulakis P, Constantinidis T, Katsiotis A, Skaracis N (2012). Ten indigenous edible plants: contemporary use in eastern Crete, Greece. Cult Agr Food Environ.

[41] Leonti M, Nebel S, Rivera D, Heinrich M (2006). Wild gathered food plants in the European Mediterranean: a comparative analysis. Econ Bot.

[42] Pieroni A, Nebel S, Quave CL, Münz H, Heinrich M (2002). Ethnopharmacology of liakra, traditional weedy vegetables of the Arbëreshë of the vulture area in southern Italy. J Ethnopharmacol.

[43] Pieroni A, Nebel S, Santoro RF, Heinrich M (2005). Food for two seasons: culinary uses of non-cultivated local vegetables and mushrooms in a south Italian village. Int J Food Sci Nutr.

[44] Nebel S, Pieroni A, Heinrich M (2006). Ta chorta: wild edible greens used in the Graecanic area in Calabria, southern Italy. Appetite.

[45] Pieroni A, Quave CL, Pieroni A, Price LL (2006). Functional foods or food-medicines? On the consumption of wild plants among Albanians and southern Italians in Lucania. Eating and healing: traditional food as medicine.

[46] Pieroni A (2001). Evaluation of the cultural significance of wild foods botanicals traditionally consumed in northwestern Tuscany, Italy. J Ethnobiol.

[47] Lentini F, Venza F (2007). Wild food plants of popular use in Sicily. J Ethnobiol Ethnomed.

[48] Pasta S, Garfì G, La Bella F, Rühl J, Carimi F. An overview on the human exploitation of Sicilian native edible plants. In: Davis RE, editor. Wild plants: identification, uses and conservation. New York: Nova Science Publishers Inc.; 2011. p. 195–268.

[49] Licata M, Tuttolomondo T, Leto C, Virga G, Bonsangue G, Cammalleri I, Gennaro MC, La Bella S (2016). A survey of wild plant species for food use in Sicily (Italy)—results of a 3-year study in four regional parks. J Ethnobiol Ethnomed.

[50] Ghirardini MP, Carli M, del Vecchio N, Rovati A, Cova O, Valigi F (2007). The importance of a taste. A comparative study on wild food plant consumption in twenty-one local communities in Italy. J Ethnobiol Ethnomed.

[51] Guarrera PM, Salerno G, Caneva G (2006). Food, flavouring and feed plant traditions in the Tyrrhenian sector of Basilicata, Italy. J Ethnobiol Ethnomed.

[52] Guarrera PM, Savo V (2013). Perceived health properties of wild and cultivated food plants in local and popular traditions of Italy: a review. J Ethnopharmacol.

[53] Ranfa A, Maurizi A, Romano B, Bodesmo M (2014). The importance of traditional uses and nutraceutical aspects of some edible wild plants in human nutrition: the case of Umbria (central Italy). Plant Biosyst.

[54] Motti R, Antiguani V, Idolo M (2009). Traditional plant use in the Phlegraean fields Regional Park (Campania, southern Italy). Hum Ecol.

[55] Guarrera PM (2003). Food medicine and minor nourishment in the folk traditions of Central Italy (Marche, Abruzzo and Latium). Fitoterapia.

[56] Sansanelli S, Tassoni A (2014). Wild food plants traditionally consumed in the area of Bologna (Emilia Romagna region, Italy). J Ethnobiol Ethnomed.

[57] Signorini MA, Piredda M, Bruschi P (2009). Plants and traditional knowledge: an ethnobotanical investigation on Monte Orbene (Nuoro, Sardinia). J Ethnobiol Ethnomed.

[58] Guarrera PM, Manzi A (2005). Wild plants of organoleptic or nutritional interest and food traditions in central Italy: some interesting cases. Plant Gen Res.

[59] Pieroni A (1999). Gathered wild food plants in the upper valley of the Serchio river (Garfagnana) Central Italy. Econ Bot.

[60] Di Novella R, Di Novella N, De Martino L, Mancini E, De Feo V (2013). Traditional plant use in the national park of Cilento and Vallo Di Diano, Campania, southern, Italy. J Ethnopharmacol.

[61] Guarrera PM, Savo V (2016). Wild food plants used in traditional vegetable mixtures in Italy. J Ethnopharmacol.

[62] Guarrera PM (2006). Usi e tradizioni della flora italiana.

[63] Menendez-Baceta G, Aceituno-Mata L, Tardío J, Reyes-García V, Pardo-de-Santayana M (2012). Wild edible plants traditionally gathered in Gorbeialdea (Biscay, Basque Country). Genet Resour Crop Evol.

[64] Pardo-de-Santayana M, Tardío J, Blanco E, Carvalho AM, Lastra JJ, San Miguel E (2007). Traditional knowledge of wild edible plants used in the northwest of the Iberian Peninsula (Spain and Portugal): a comparative study. J Ethnobiol Ethnomed.

[65] Pardo-De-Santayana M, Tardío J, Morales R (2005). The gathering and consumption of wild edible plants in the Campoo (Cantabria, Spain). Int J Food Sci Nutr.

[66] Tardío J, Pascual H, Morales R (2005). Wild food plants traditionally used in the province of Madrid, Central Spain. Econ Bot.

[67] Bonet MA, Vallès J (2002). Use of non-crop food vascular plants in Montseny biosphere reserve (Catalonia, Iberian Peninsula). Int J Food Sci Nutr.

[68] Tardío J, Pardo-de-Santayana M, Morales R (2006). Ethnobotanical review of wild edible plants in Spain. Bot J Linn Soc.

[69] González JA, García-Barriuso M, Amich F (2011). The consumption of wild and semi-domesticated edible plants in the Arribes del Duero (Salamanca-Zamora, Spain): an analysis of traditional knowledge. Genet Res Crop Evol.

[70] Rigat M, Gras A, D’Ambrosio U, Garnatje T, Parada M, Vallès J (2016). Wild food plants and minor crops in the Ripollès district (Catalonia, Iberian Peninsula): potentialities for developing a local production, consumption and exchange program. J Ethnobiol Ethnomed.

[71] Rivera D, Obón C, Inocencio C, Heinrich M, Verde A, Fajardo J, Palazón JA (2007). Gathered food plants in the mountains of Castilla-La Mancha (Spain): ethnobotany and multivariate analysis. Econ Bot.

[72] Sánchez-Mata MC, Loera RDC, Morales P, Fernández-Ruiz V, Cámara M, Marqués CD (2012). Wild vegetables of the Mediterranean area as valuable sources of bioactive compounds. Genet Resour Crop Evol.

[73] Nassif F, Tanji A (2013). Gathered food plants in Morocco: the long forgotten species in ethnobotanical research. Life Sci Leafl.

[74] Powell B, Ouarghidi A, Johns T, Tattou MI, Eyzaguirre P (2014). Wild leafy vegetable use and knowledge across multiple sites in Morocco: a case study for transmission of local knowledge?. J Ethnobiol Ethnomed.

[75] Schicchi R, Geraci A (2015). Verdure spontanee di Sicilia.

[76] Lentini F, Catanzaro F, Aleo M (1988). Indagini etnobotaniche in Sicilia. III. L'uso tradizionale delle piante nel territorio di Mazara del Vallo (Trapani). Atti Accad Sci Lett Arti Palermo..

[77] Raimondo FM, Lentini F (1990). Indagini etnobotaniche in Sicilia. I. Le piante della flora locale nella tradizione popolare delle Madonie (Palermo). Naturalista Sicil.

[78] Lentini F, Raimondo FM (1990). Indagini etnobotaniche in Sicilia. IV. L'uso tradizionale delle piante nel territorio di Mistretta (Messina). Quad Bot Amb Appl.

[79] Lentini F, Aleo M (1991). Indagini etnobotaniche in Sicilia. V. L'uso tradizionale delle piante nel territorio di Erice (Trapani). Atti Accad Sci Lett Arti Palermo.

[80] Ilardi V, Raimondo FM (1992). L’uso tradizionale delle piante nella comunità rurale di Mezzojuso (Palermo). Quad Bot Amb Appl.

[81] Lentini F, Giani S, Amenta R (1995). L'uso popolare delle piante nelle isole Eolie (Sicilia). Acta technol legis medicament.

[82] Lentini F, Di Martino A, Amenta R (1994). La flora popolare di Ustica (Palermo). Giorn Bot Ital.

[83] Lentini F, Di Martino A, Amenta R (1995). Le piante di uso popolare nell'arcipelago delle Pelagie (Ag). L'uomo e l'ambiente.

[84] Lentini F, Aleo M, Amenta R (1997). L'uso popolare delle piante nelle Isole Egadi (Sicilia). Acta Phytoterap.

[85] Lentini F (1996). Gli usi tradizionali delle piante di Sant'Angelo Muxaro. Atti del Convegno" Natura, Mito & Storia nel Regno Sicano di Kokalos".

[86] Catanzaro F (2002). Note sulle piante di uso popolare dei territori di Pantelleria e Bivona (Ag). Etnobotanica nella Provincia di Catania con Atti del Convegno "Andar per verdure".

[87] Lentini F (2002). L'etnobotanica in Sicilia: le piante alimentari di uso popolare. Etnobotanica nella Provincia di Catania con Atti del Convegno "Andar per verdure".

[88] Arcidiacono S, Pavone P (1994). Erbe spontanee commestibili del territorio etneo. Boll Acc Gioenia Sci Nat (Catania).

[89] Arcidiacono S (2002). Flora popolare nel territorio di Bronte (CT). Etnobotanica nella Provincia di Catania con Atti del Convegno "Andar per verdure".

[90] Arcidiacono S (1998). Le verdure spontanee dell'Etna.

[91] Arcidiacono S, Pavone S. Le piante alimurgiche. Le erbe spontanee commestibili del territorio Etneo. last update: 13/4/2007, [http://www.dipbot.unict.it/alimurgiche]. Accessed 20 Apr 2017.

[92] Napoli M (2002). Usi popolari di Barlia robertiana (Loisel.) W. Greut. (Orchidaceae) nel territorio di Santo Pietro di Caltagirone (Catania). Etnobotanica nella Provincia di Catania con Atti del Convegno "Andar per verdure".

[93] Arcidiacono S, Pavone P, Napoli M (2003). Piante spontanee d’uso popolare nel territorio di Bronte (Catania). Quad Bot Amb Appl.

[94] Arcidiacono S, Napoli M, Oddo G, Pavone P (2007). Piante selvatiche d’uso popolare nei territori di Alcara Li Fusi e Militello Rosamarino (Messina). Quad Bot Amb Appl.

[95] Arcidiacono S, Costa R, Marletta G, Pavone P, Napoli M (2010). Usi popolari delle piante selvatiche nel territorio di Villarosa (EN – Sicilia Centrale). Quad Bot Amb Appl.

[96] Arcidiacono S, Pavone P, Salmeri C. Le piante alimurgiche [Internet]. Le piante spontanee di uso alimentare nel Territorio Etneo. 2005; Available from: http://www.dipbot.unict.it/alimurgiche/introduzione.htm. cited 1 Apr 2015.

[97] Lucchesi T (2004). Piano Stralcio di Bacino per l’Assetto Idrogeologico della Regione Siciliana. Relazione generale. Regione Siciliana. Assessorato Territorio e Ambiente.

[98] Bazan G, Marino P, Guarino R, Domina G, Schicchi R (2015). Bioclimatology and vegetation series in Sicily: a geostatistical approach. Ann Bot Fenn.

[99] Medail F, Quezel P (1997). Hot-spots analysis for conservation of plant biodiversity in the Mediterranean Basin. Ann Mo Bot Gard.

[100] Raimondo FM, Domina G, Spadaro V (2010). Checklist of the vascular flora in Sicily. Quad Bot Amb Appl.

[101] Gianguzzi L, Papini F, Cusimano D (2016). Phytosociological survey vegetation map of Sicily (Mediterranean region). J Maps.

[102] Baiamonte G, Domina G, Raimondo FM, Bazan G (2015). Agricultural landscapes and biodiversity conservation: a case study in Sicily (Italy). Biodivers Conserv.

[103] ISE International Society of Ethnobiology Code of Ethics (with 2008 additions). 2006. http://ethnobiology.net/code-of-ethics/. Accessed 20 Feb 2017.

[104] Pignatti S. Flora d’Italia. Bologna: Edagricole; 2003.

[105] The Plant List – version 1. 2013. http://www.theplantlist.org. Accessed 10 Apr 2017.

[106] Conti F, Abbate G, Alessandrini A, Blasi C (2005). An annotated checklist of the Italian vascular flora.

[107] Giardina G, Raimondo FM, Spadaro V (2007). A catalogue of plants growing in Sicily. Bocconea.

[108] Fici S (2014). A taxonomic revision of the *Capparis spinosa* group (Capparaceae) from the Mediterranean to Central Asia. Phytotaxa.

[109] Fici S (2015). A taxonomic revision of the *Capparis spinosa* group (Capparaceae) from eastern Africa to Oceania. Phytotaxa.

[110] Raunkiaer C (1937). Plant life forms.

[111] McGarigal K, Cushman SA, Stafford S. Multivariate statistics for wildlife and ecology research. New York: Springer Science & Business Media; 2013.

[112] Podani J. SIN-TAX 2000, computer programs for multivariate data anlysis in ecological sistematics. Budapest: Scientia Publishing; 2001.

[113] Bulgarelli G, Flamigni S (2010). Le piante tossiche e velenose.

[114] Turner NJ, Luczaj L, Migliorini P, Deon AL, Sacchetti LE, Paoletti MG (2011). Edible and tended wild plants, traditional ecological knowledge and agroecology. Crit Rev Plant Sci.

[115] Castroviejo S, Aedo C, Cirujano S, Laínz M, Montserrat P, Morales R, Muñoz Garmendia F, Navarro C, Paiva J, Soriano C (1986). Flora Ibérica. Plantas vasculares de la Península Ibérica e Islas Baleares.

[116] Güner A, Özhatay N, Ekim T, Başer KHC (2000). Flora of Turkey and the East Aegean Islands.

[117] Alzweiri M, Al-Shudeifat M, Al-Khaldi K, Al-Hiari Y, Afifi FU (2015). Acetylated ferulenol-oxy-ferulenol as a proposed marker for fresh Ferula toxicity: a metabolomics approach. J Liq Chromatogr Relat Technol.

[118] Akaberi M, Iranshahy M, Iranshahi M (2015). Review of the traditional uses, phytochemistry, pharmacology and toxicology of giant fennel (*Ferula communis* L. subsp. *communis*). Iran J Basic Med Sci.

[119] Rivera D, Obón C, Heinrich M, Inocencio C, Verde A, Fajardo J. Gathered Mediterranean food plants—ethnobotanical investigations and historical development. In: Heinrich M, Müller WE, Galli C, editors. Local Mediterranean Food Plants and Nutraceuticals. Forum Nutr. Basel: Karger, 2006;59:18–74. 10.1159/000095207 16917173

[120] Atzei AD (2003). Le piante nella tradizione popolare della Sardegna.

[121] Benhouda A, Yahia M (2015). Toxicity and anti-inflammatory effects of methanolic extract of *Umbilicus rupestris* L. leave (crassulaceae). Int J Pharm Bio Sci.

